# Biotransformation of As, Cr, Hg, and Mn by Pseudomonadota: chances and risks

**DOI:** 10.1007/s10532-025-10157-x

**Published:** 2025-07-15

**Authors:** Aleksandra Naziębło, Jakub Dobrzyński

**Affiliations:** Institute of Technology and Life Sciences – State Research Institute, Raszyn, Poland

**Keywords:** Soil bacteria, Pseudomonadota, Heavy metals, Redox transformation, Bioremediation

## Abstract

Heavy metals are among the key environmental contaminants which pose a threat to all the living organisms. Bacteria can help detoxify these elements by a range of mechanisms associated to bacterial metabolic processes. One of them is biotransformation, which consists in change in the oxidative state of an element, often followed by its absorption or precipitation. Pseudomonadota—a vast and diverse bacterial phylum—belong to the best studied microorganisms involved in the process. Our review shows their contribution to the redox-based detoxification of four trace elements: chromium, manganese, mercury, and arsenic. Based on a comprehensive literature survey, we try to assess the importance of individual orders and selected genera in redox transformations; we also identify potential risks associated with the use of Pseudomonadota in bioremediation and suggest the most promising directions for future studies and applications. The review leads to a conclusion that the potential of non-pathogenic rhizobia Hyphomicrobiales is particularly worth further exploration.

**Supplementary Information:**

The online version contains supplementary material available at 10.1007/s10532-025-10157-x.

## Introduction

Heavy metals are among the key environmental contaminants. They are introduced to soil and water through various forms of human activity such as mining, metal industry, fuel combustion, intensive agriculture, and improper waste management (Gamalero et al. 2009; Briffa et al. 2020; Wróbel et al. 2023). Once accumulated in the environment, heavy metals become a serious threat as they are very stable and do not undergo biodegradation or decay (Aryal and Aryal and Liakopoulou-Kyriakides 2015; Briffa et al. 2020). They can be, however, absorbed by microorganisms, animals, and plants. Toxic ions accumulate in tissues of all organisms, posing a threat to their consumers including people (Puschenreiter et al. 2005; Komal et al. 2015). In vertebrates, heavy metals cause numerous disorders, such as impairment of central nervous system and circulatory system, internal organ damage, respiratory failure, and cancer (Kang et al. 2007; Tchounwou et al. 2012). Hence, heavy metal contamination affects not only the natural environment but also economics, public health, or food policy.

Bacteria are able to transform or immobilise heavy metals, making them less available or less toxic to plants and animals. The main mechanisms by which they sequester harmful elements are biosorption and bioprecipitation (Valls and de Lorenzo 2002; Aryal and Liakopoulou-Kyriakides 2015; Barra Caracciolo and Terenzi 2021). In addition to the direct removal of toxic ions from the environment, bacteria and their metabolites can reduce the harmful effects of plant exposure to heavy metals. Some microorganisms moderate the toxicity of metals by modifying their redox state, while others affect the plant defense system. Numerous substances produced by bacteria participate in the regulation of organisms’ response to stress and the alleviation of its effects (Etesami 2018; Barra Caracciolo and Terenzi 2021).

One of the most beneficial features found in heavy metal-detoxifying bacteria is the ability to induce redox processes. Microorganisms not only contribute to the sequestration of toxic ions, but can also directly influence the metal oxidation state. Some trace elements occur in various redox forms, differing in mobility and toxicity (Malaviya and Singh 2016). These include chromium, mercury, manganese, and arsenic—a metalloid, traditionally incorporated into the group of heavy metals on a toxicity basis. By transforming a metal into a less harmful form, bacteria help detoxify it. At times, a change in oxidation state is required to make an element more susceptible to adsorption or precipitation processes.

**Fig. 1:**
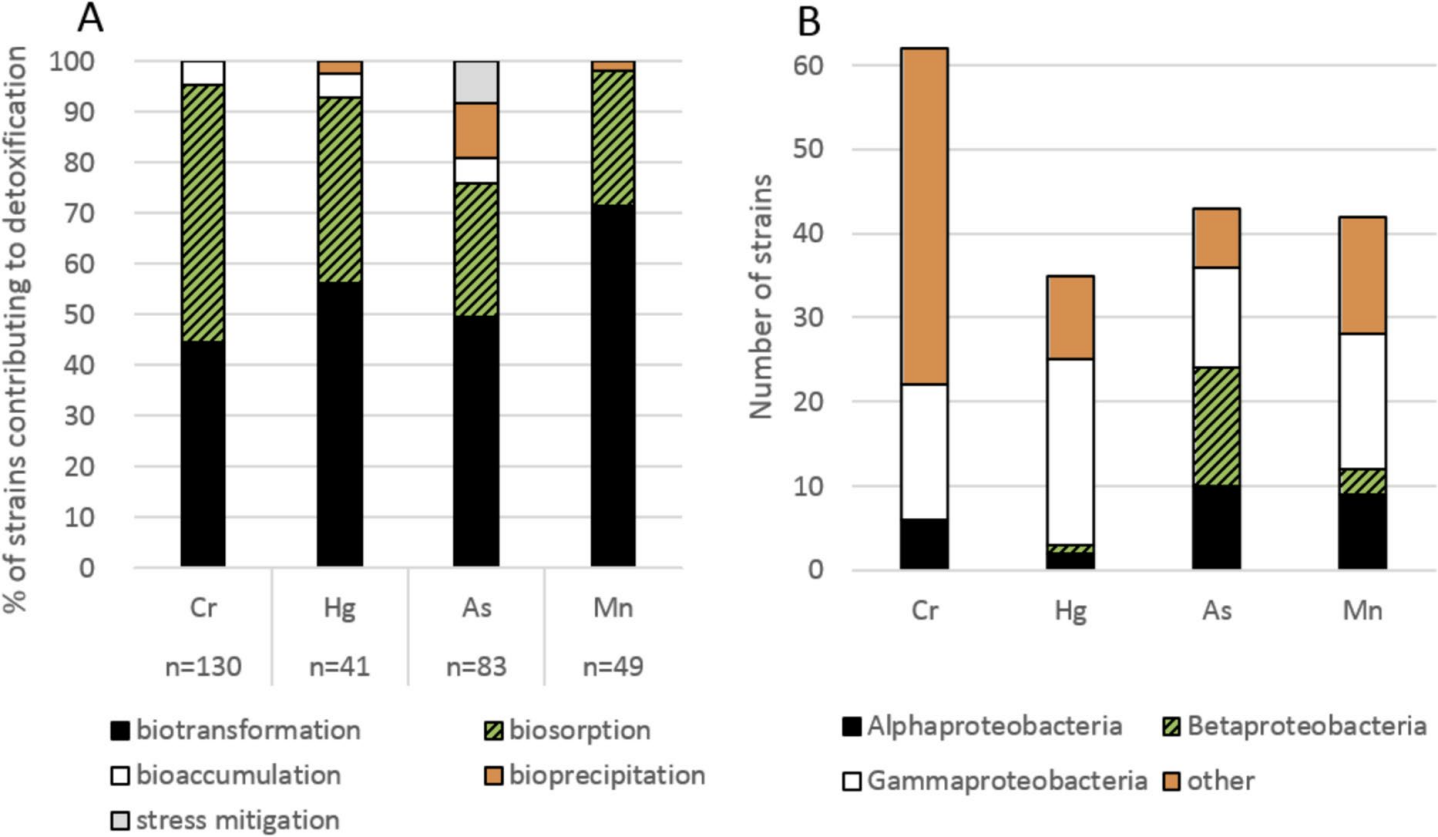
Biotransformation of chromium, mercury, arsenic, and manganese. **A** Contribution of different microbial-induced processes to the detoxification of four elements; **B** involvement of Pseudomonadota in the redox transformation of each element (list of references in Online Resource S1)

Redox transformation belongs to the most important mechanisms of the microbial-induced detoxification of chromium, mercury, manganese, and arsenic (Fig. 1A), and the phylum Pseudomonadota contributes significantly to this process (Fig. 1B). The three former elements are mostly reduced or oxidised by Alpha-, Beta-, and Gammaproteobacteria (60–80% of all bacteria participating in biotransformation). In addition, almost one third of strains reported to perform chromate reduction belong to Pseudomonadota.

Our review aims at summarising the role of Pseudomonadota in the detoxification of Mn(II), Hg(II), As(III), and Cr(VI) into their less harmful forms. We describe the mechanisms underlying the biotransformation processes, characterise redox abilities of each class and the contribution of some important genera to heavy metal detoxification. Finally, we analyse the risk associated with the use of potentially pathogenic bacterial strains and identify the most promising direction for further investigations.

Throughout the text, we use some terms that have similar meaning and might be easily confused, such as "detoxification", “bioremediation”, and "biotransformation". We decided to define them here to avoid misunderstanding. By detoxification we mean any process that minimises the impact of contaminants on living organisms. This includes both toxicity mitigation and reduction of bioavailability. Bioremediation refers to any technique that employs organisms for detoxification or removal of pollutants. Finally, biotransformation is a process in which living organism converts the chemical compound into another form; here we refer to the ability of microorganisms to modify the oxidative form of heavy metals.

## Mechanisms of biotransformation

**Fig. 2:**
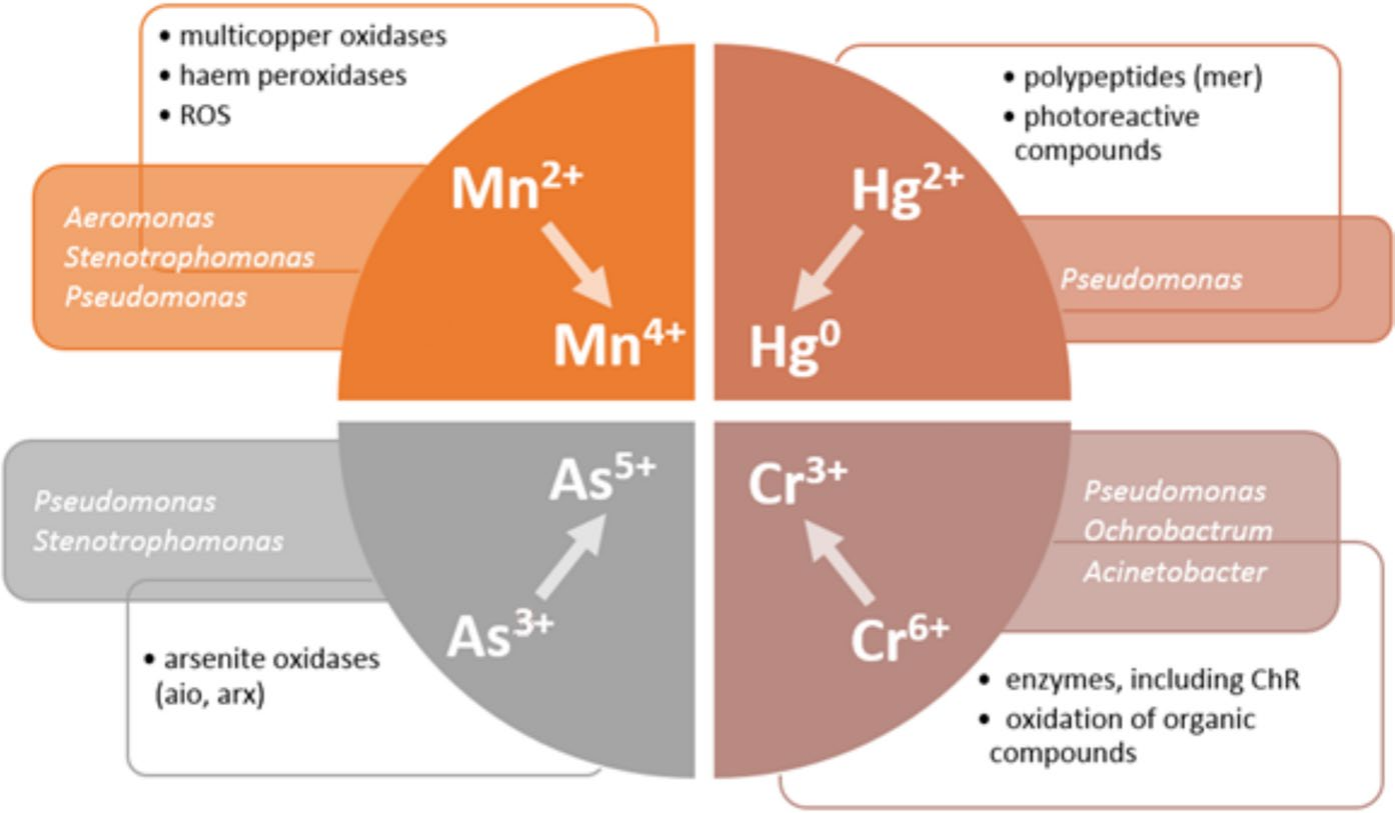
Biotransformation of chromium, mercury, arsenic, and manganese. **A** Contribution of different microbial-induced processes to the detoxification of four elements; **B** involvement of Pseudomonadota in the redox transformation of each element

Many different types of metabolic activity are involved in the biotransformation of trace elements. The change in metal valence may help bacteria overcome the oxidative stress, but it may also be a side effect of other important processes such as respiration or energy acquisition (Piazza et al. 2019; Yao et al. 2020; Yu et al. 2020). Microorganisms synthesise a range of oxidoreductases that transform heavy metals into less toxic forms. They use both organic and inorganic compounds as electron donors or acceptors (Pradhan et al. 2016; Wang et al. 2022; Biełło et al. 2023). The most important mechanisms leading to the biotransformation of four trace elements are presented in Fig. 2, along with the main genera of Pseudomonadota involved in the processes.

### Chromium

Two relatively stable chromium forms are most common in the environment: Cr(III) and Cr(VI). The first one occurs naturally in various insoluble complexes such as hydroxides, oxides, and sulphates; the second one, originating mainly from anthropogenic sources, is soluble and persistent (Ahemad 2014). Chromates are also highly toxic, mutagenic, and carcinogenic as they easily penetrate through biological membranes and can interact with DNA or proteins (Sagar et al. 2012; Malaviya and Singh 2016; Hu et al. 2021). Intracellular Cr^3+^ ions exert harmful effects too; they are able to inhibit DNA replication and react with functional groups in enzymes (Ahemad 2014). However, due to the low solubility of chromium (III) precipitates, only a small amount of toxic ions can pass through the cell membrane (Hu et al. 2021). For this reason, trivalent chromium is considered a thousand times less noxious than its hexavalent form (He et al. 2009; Malaviya and Singh 2016; Hu et al. 2021).

Microorganisms cope with toxic Cr(VI) through uptake limitation, ion exclusion using chromium-induced efflux pumps, and biosorption (González et al. 2014; Banerjee et al. 2019). Numerous bacteria tend to reduce the metal to a less harmful form before they use other processes, such as biosorption or bioaccumulation. Chromate-reducing organisms act in two ways: they harness soluble or membrane-associated enzymes to reduce Cr(VI) directly or form complexes with metabolites and use them as electron donors (Sagar et al. 2012; Shi et al. 2012; Tan et al. 2020). In anaerobes, electrons from the respiratory chain may also act as reductants for chromates (Alam and Malik 2008; Yao et al. 2020).

A number of carbon sources can be oxidised in order to reduce Cr(VI)—amino acids, nucleotides, organic acids (pyruvate, citrate, formate, and lactate), carbohydrates (lactose, fructose, and glucose), vitamins, glutathione, flavo- and hemeproteins, NADH, and NADPH (Shi et al. 2012; Ahemad 2014). Iron- and sulphate-reducing bacteria are also able to use the end-products of their metabolism (Fe^2+^ ions or hydrosulfide ions) as reductants (Malaviya and Singh 2016; Yao et al. 2020).

Several bacterial enzymes occurring in chromium resistant bacteria have been identified as chromate reductases belonging to groups such as cytochromes, hydrogenases, and flavin reductases (Thatoi et al. 2014). A chromate reductase ChrR can be found in a number of gammaproteobacterial strains, including *Pseudomonas putida*, *Echerichia coli*, and *Stenotrophomonas* sp.; *E. coli* has also been shown to utilise other flavoproteins (YieF, NemA) as Cr(VI) reductases (Thatoi et al. 2014; Ge and Ge 2016). However, most enzymes involved in the process are not exclusively bound to chromate reduction and usually participate in the reduction of other ions and compounds, including other metals (Opperman and Van Heerden 2007; Ahemad 2014; Yao et al. 2020). Depending on the reductase localization, the process may take place intra- or extracellularly, which affects the further fate of metal ions (Opperman and Van Heerden 2007; Tan et al. 2020).

Reduced chromium ions are released into the medium (Banerjee et al. 2019), adsorbed on the cell surface (Mondaca et al. 2002; Garg et al. 2013), or accumulated inside the cells (Cheung et al. 2006). In numerous studies, both extracellular and intracellular sequestration of Cr(III) ions was observed (Batool 2012; González et al. 2014; Tan et al. 2020). Many microorganisms belonging to Pseudomonadota cope with Cr(VI) stress both reducing and immobilising toxic ions (Table 1).

**Table 1:** Cr(VI) reduction by Pseudomonadota

Order	Genus/species	Isolation site	Initial Cr concentration [mM]	Cr removal efficiency	Suggested oxidation mechanism	Additional detoxification mechanisms	References
Hyphomicrobiales	*Ochrobactrum* sp.	Chromium landfill	3.85–15.39	75–100%/48 h			He et al. (2009)
	*O. intermedium*	Tannery effluent	1.9–19.2	90.8–96.1%/72 h	Enzymatic pathway	Adsorption, accumulation	Batool (2012)
	*O. intermedium*	Chromium polluted soil	2.15	100%/72 h	Quinone reductase and Cr reductase		Kavitha and Keharia (2012)
	*Brucella* sp.	Dyestuff industrial area	0.96–3.85	45–100%/54 h	Cr reductase		Thacker et al. (2007)
	*Pannonibacter phragmitetus*	Fish pond	19.2	65%/15 h	Enzymatic pathway		Shi et al. (2012)
Lysobacterales	*Stenotrophomonas* sp.	Seawater near electroplating and tannery factories	1.9–13.5	25–100%/48 h	Cr reductase (chrR)		Ge and Ge (2016)
	*S. maltophilia*	Wastewater	0.3–9.6	60–100%/48 h	Cr reductase (chrR)		Baldiris et al. (2018)
	*S. rhizophila*	Rapeseed rhizosphere	0.96	100%/28 h	FMN-dependent NADH-azoreductase; flavin reductase	PGPB, adsorption, accumulation	Gao et al. (2020)
	*Stenotrophomonas*	Tannery—soil	1.06	80%/6 h			Gunasundari and Muthukumar (2013)
	*S. maltophilia*		0.25–0.5	60–100%/24 h		Sorption	Alam and Ahmad (2012)
Enterobacterales	*Serratia* sp.	Iron ore mines	0.08–0.38	85–100%/24 h		Adsorption	Upadhyay et al. (2020)
	*Serratia* sp.	Effluents and sediments from a tannery	0.096–1.92	16–75%/120 h (including biosorption)	Cr reductase	Adsorption, accumulation	González et al. (2014)
	*S. marcescens*	Tannery effluent	0.45	> 75%/48 h		Biofilm formation	Mondaca et al. (2002)
Pseudomonadales	*P. umsongensis*	Cr-contaminated soil	0.096–0.865	11–80%/24 h	Cr reductase (chrR)		Yao et al. (2020)
	*P. entomophila*	Cr-cont. Effluent	1.92	86%/120 h	Cr reductase	PGPB	Wani et al. (2019)
	*Pseudomonas*	Rapeseed rhizosphere	0.19–0.38	56.5- 84.3%/ 48 h	Cr reductase (chrR); upregulated azoreductase (azoR)		Shi et al. (2023)
	*P. aeruginosa*	Tannery effluent	0.31–0.77	45–100%/30 h			Ganguli and Tripathi (1999)
	*P. fluorescens*	Cr contaminated river sediment		50%/48 h	Cr reductase		Bopp and Ehrlich (1988)
	*P. aeruginosa*	Textile industrial wastewater	0.19	> 85%/16 h		Adsorption and accumulation	Ozturk et al. (2012)
	*P. aeruginosa*		0.19	> 60%/16 h			Ozturk et al. (2012)
	*P. stutzeri*	Groundwater aquifer solids	0.01–0.025	60–80%/48 h	Siderophore pyridine-2,6-bis(thiocarboxylic acid)	Siderophores, precipitation	Zawadzka et al. (2007)
	*P. stutzeri*	Crude oil	1.9–19	40–97%/24 h			Sathishkumar et al. (2017)
	*P. putida*	Dairy sludge	9.62	82%/72 h (80% reduction)		Adsorption	Garg et al. (2013)
	*P. aeruginosa*	Landfill leachate treating reactor	0.096	80%/12 h (70% reduction)	Cr reductase		He et al. (2015)
Moraxellales	*Acinetobacter* sp.	Heavy metal-rich water	0.135–0.4	78–100%/24 h		Adsorption, accumulation	Bhattacharya and Gupta (2013)
	*A. indicus*	Cr-contaminated soil	0.385–1.154	77–93.5%/ 60 h	FMN reductases and nitroreductase; upregulation of *chr*A gene		Hu et al. (2021)
	*A. haemolyticus*	Textile dye effluent	0.19–1.9	21–67%/48 h	Cr reductase	Adsorption (carboxyl, hydroxyl or amide groups)	Ahmad et al. (2013)
	*A. junii*	Chromite mine	1.04	99%/12 h			Pulimi (2012)
	*Acinetobacter* sp.	Tannery effluent	0.96–3.85	56.875–100%/72 h		Accumulation	Essahale et al. (2012)
	*A. radioresistens*	Soil	1.154–3.08	> 30–100%/48 h			Ram Talib et al. (2019)
	*A.calcoaceticus*		1.9	> 85%/24 h			Samantaray and Mishra (2012)
	*A. baumannii*	Crude oil	1.9–19	84–99%/24 h			Sathishkumar et al. (2017)
	*Acinetobacter* sp.	Dye effluent treatment facility	3.85	100%/78 h			Narayani and Vidya Shetty (2012)

The chromate reduction rate is significantly affected by the presence of other metals in the solution. The stimulating role of copper is frequently reported (Sarangi and Krishnan 2008; He et al. 2009; Sayel et al. 2012; Min et al. 2024); other metals considered beneficial for the reduction process are cobalt and manganese (He et al. 2009; Shi et al. 2012; Ibrahim et al. 2012). On the other hand, nickel and cadmium seem to decrease the reduction rate (Pal et al. 2005; Shi et al. 2012). Some microorganisms demonstrate the ability to reduce chromium together with other ions such as Se(IV), Se(VI), As(V), or Hg(II) (Cheung et al. 2006; Yao et al. 2020).

### Mercury

Mercury, one of the most toxic elements in the environment, occurs in three forms: elemental, inorganic, and organic (Jan et al. 2009). Although metallic mercury is highly toxic, it is less harmful to living organisms than its monovalent or divalent forms (Chien et al. 2012; Dash et al. 2017). Organic compounds, formed with Hg(II) bound to methyl or phenyl groups, exhibit the highest toxicity since they easily cross biological membranes; however, both organic and inorganic mercury forms attach to sulfhydryl or disulfide groups of enzymes and proteins, thus disturbing their action (Jan et al. 2009; Mahbub et al. 2016a; Hu et al. 2021).

The bioremediation of mercury-polluted soils is mainly based on the reduction of Hg(II) into a less toxic elemental form (Mahbub et al. 2016b; Franco et al. 2018). The process is also referred to as volatilisation because reduced mercury becomes volatile. It is mediated by a mercury resistance (mer) operon encoding polypeptides responsible for mercury transport and transformation (Osborn et al. 1997; Yao et al. 2020). The operon may be induced or suppressed, depending on the presence of mercury in the environment (Giovanella et al. 2016). Several proteins participate in the detoxification process, including merP (initial ion sequestration in the periplasm by binding to cysteine thiol groups), merT (transport into the cytoplasm), and merA (enzymatic reduction mediated by a flavoprotein—mercuric reductase) (Osborn et al. 1997; Franco et al. 2018). The conversion of Hg(II) into its elemental form, catalysed by mercuric reductase, requires NADPH and FAD as electron donors (Dash et al. 2017). Two other enzymes—glutaredoxin 1 and carboxylesterase E2—are responsible for maintaining the flavoprotein in a reduced form, thus indirectly participating in the volatilisation (Dash et al 2017). Organomercurial compounds are degraded with the involvement of organomercury lyase (merB) before the subsequent redox transformation (Franco et al. 2018). In addition, regulatory genes (*mer*R and *mer*D), a resistance gene *mer*G, and other genes probably also assist in the transport through the membranes—*mer*C, *mer*E, *mer*F, and *mer*H (Jan et al. 2009; Yao et al. 2020). Some studies show that engineered microorganisms, provided with *mer* genes, adsorb mercury more effectively than wild-type strains, while transgenic plants are even capable of converting it into less toxic elemental form (Bae et al. 2003; Bolan et al. 2014).

Most mercury-volatilising bacteria possess the *mer*A gene encoding Hg(II) reductase (Table 2). However, some bacterial strains show the ability to reduce Hg(II), although the transformation is not related to *mer*A activity (Wiatrowski et al. 2006; De et al. 2008). In the case of *Shewanella oneidensis* tested by Wiatrowski et al. (2006), the process required additional compounds serving as electron acceptors and donors, which suggested an involvement of respiratory electron transport chains. Similarly, Fe(II)—dependent mercury volatilisation has been observed in *Acidithiobacillus ferrooxidans* cultured by Sugio et al. (2003). On the other hand, phototrophic bacteria can perform the conversion of Hg(II) into elemental mercury through the photoreactive compounds formed during photosynthesis (Franco et al. 2018). In this case the volatilisation occurs under the influence of UV light absorbed by mercury compounds (Franco et al. 2018). However, no such mechanism has been observed to date in Pseudomonadota.

**Table 2:** Hg(II) reduction by Pseudomonadota

Order	Species	Isolation site	Initial Hg concentration [µM]	Hg removal efficiency	Suggested oxidation mechanism	Additional detoxification mechanisms	References
Hyphomicrobiales	*Xanthobacter autotrophicus*	Soil	0.2	90.2%/7.5 h	Hg reductase (merA)		Petrus et al. (2015)
Sphingomonadales	*Sphingobium* sp.	Hg contaminated site	16	79%/6 h	Hg reductase (merA)		Mahbub et al. (2016a, b)
Lysobacterales	*Pseudoxanthomonas* sp.	Hg contaminated soil	7	60%/6 h	Hg reductase (merA)	Accumulation	Mahbub et al. (2016a, b)
Vibrionales	*Vibrio* sp.	Seawater	50	ca. 70%/48 h	Hg reductase (merA)		Dash et al. (2017)
Enterobacterales	*Citrobacter freundii*	Arctic fjord water	25	80%/240 h		Sorption	Binish et al. (2021)
	*Escherichia coli*	Seawater	50	65.78%/48 h	Hg reductase (merA)		Dash et al. (2017)
	*Klebsiella pneumoniae*	Wastewater	250	> 30%/20 h			Zeroual et al. (2001)
Pseudomonadales	*P. umsongensis*	Cr-contaminated soil2	25–224	ca. 25%- ca. 70%/24 h	Hg reductase (merA)		Yao et al. (2020)
	*P. putida*	Pond of cinnabar mine wastes	5	ca. 80%/ 180 h		Accumulation	Baldi et al. (1993)
	*P. putida*	Landspreading areas used to treat Hg-contaminated petrochemical waste	10–350	54.30–83.67%/24 h	Hg reductase (merA)		Giovanella et al. (2016)
	*Pseudomonas* sp.	Landspreading areas used to treat Hg-contaminated petrochemical waste	10–350	51.08–82.24%/24 h	Hg reductase (merA)		Giovanella et al. (2016)
	*P. putida*	Seawater	280	ca. 80–100%/48 h	Hg reductase (merA) and Hg lyase (merB)		Zhang et al. (2012)
	*P. aeruginosa*	Seawater	50	ca. 95%/48 h	Hg reductase (merA)		Dash et al. (2017)
	*P. aeruginosa*	Clinical sewage	10–50	80–100%/3 h			Chang and Law (1998)
Alteromonadales	*Shewanella oneidensis*	Laboratory culture (spontaneous mutant)	0.15	65%/24 h	Activity of respiratory electron transport chains	Sorption	Wiatrowski et al. (2006)
Moraxellales	*Acinetobacter indicus*	Cr-contaminated soil	50–150	ca. 80–100%/48 h	merA, merB, merC, merT, merP		Hu et al. (2021)
Acidithiobacillales	*Acidithiobacillus ferrooxidans*	Laboratory culture of *A. thiobacillus*	5	95%/96 h	Fe(II) oxidation, cytochrome c oxidase		Sugio et al. (2003)

The reduced, metallic form of mercury is volatile and poorly soluble in water. Also, before being deposited, it is able to travel a long distance in the air as a vapour (Wiatrowski et al. 2006; Chien et al. 2012; Chen et al. 2019). After entering water, metallic mercury can be easily methylated, thereby posing another threat to the environment (Von Canstein et al. 2002). However, it is possible to sequester the volatilised element using absorbents or a bioreactor (Mahbub et al. 2016b; Chen et al. 2019). Depending on environmental conditions, elemental mercury can be bound to the cell surface components, retained by exopolymers, phytochelatins, or glutathione, or precipitated in the form of HgS (cinnabar) (Mahbub et al. 2016b; Franco et al. 2018; Chen et al. 2019). Although the bioprecipitation of mercury compounds is a rare phenomenon compared to biosorption, it should be noted that in anaerobic conditions mercury sulphide is readily methylated by sulphate reducing bacteria (Mahbub et al. 2016b).

### Manganese

Manganese is an essential micronutrient in plant and animal organisms, and plays a coenzyme role in several biochemical processes (Millaleo et al. 2010; Wu et al. 2022b). However, excessive metal accumulation in the tissues leads to oxidative stress and poisoning (Wang et al. 2009; Queiroz et al. 2018; Piazza et al. 2019). Three manganese oxidation states are common in biological systems (II, III, and IV), but only Mn(II) is highly soluble and readily available for plants (Millaleo et al. 2010; Nguyen et al. 2020). Therefore, the detoxification of manganese requires its oxidation to insoluble forms.

A number of microorganisms found in diverse environments have been shown to oxidise divalent manganese ions (Queiroz et al. 2018; Piazza et al. 2019). The ability to oxidise Mn(II) is a way to acquire energy in chemolithoautotrophs (Yu and Leadbetter 2020); it is also possible that it helps neutralise the toxicity of hydrogen peroxide (Piazza et al. 2019). The microbial-induced oxidation induced by bacteria and fungi is much faster than the naturally occurring chemical processes, and is believed to be the main source of manganese oxides in the environment (Tang et al. 2016).

The best understood mechanism of microbial Mn(II) oxidation involves the participation of multicopper oxidases (e.g. MnxG, McoA, and MofA) and haem peroxidases (e.g. Mn peroxidase and lignin degradation enzymes) (Andeer et al. 2015; Tang et al. 2016; Zhao et al. 2019; Wu et al. 2022b). The activity of enzymes is usually directed at the outer cell surface, causing the biogenic oxides to adhere to the cell wall (Piazza et al. 2019). However, apart from the direct enzyme-mediated biooxidation pathway, there are also indirect processes involved in the transformation of Mn(II). These consist in the modification of redox conditions through the production of reactive oxygen species and a rise in pH (Zhao et al. 2019; An et al. 2021). Direct and indirect pathways may occur both separately and simultaneously (Wu et al. 2022a). For example, Andeer et al. (2015) reported that a marine bacterium *Roseobacter* sp. performs indirect manganese oxidation which involves superoxide generation mediated by haem peroxidase. Yang et al. (2023) argue that superoxide production is the main mechanism responsible for bacterial manganese oxidation. According to the authors, heterotrophic microorganisms generate superoxide in response to light irradiation, independently of manganese presence, and the oxidation of Mn(II) could be seen as a side effect.

Oxidised manganese usually precipitates as (oxy)(hydr)oxides. Besides, Mn(III) can also form complexes with organic compounds or disproportionate to more stable oxidation states—Mn(II) and Mn(IV) (Andeer et al. 2015; Tang et al. 2016; Cai et al. 2023). Moreover, it is possible that the precipitates exhibit an intermediate valence (Zhao et al. 2019).

Apart from transforming Mn(II) into insoluble—and thus less toxic—Mn(III) and Mn(IV) forms, the manganese oxidising bacteria also exhibit the ability to adsorb and accumulate manganese. In addition, some microorganisms demonstrate plant growth-promoting potential (Li et al. 2022). The large surface area to volume ratio of bacterial cells ensures a significant contact area to interact with metal ions. Macromolecular compounds present in the cell wall, such as proteins and polysaccharides, adsorb manganese and thus immobilise it (Queiroz et al. 2018; An et al. 2021). Furthermore, the precipitated oxides act as potent sorbents for Mn(II). Liao et al. (2013), who tested the bioremediation potential of a deep-sea *Marinobacter* bacterium, found that 76,4% of Mn(II) underwent microbial-induced oxidation, while 23,4% was absorbed by the generated (hydr)oxides; the total manganese removal amounted to 99,9%.

The biogenic manganese oxides not only facilitate the immobilisation of toxic Mn(II) but also contribute to the sequestration of other heavy metals through absorption and oxidation (Toyoda and Tebo 2013; Andeer et al. 2015; Zhao et al. 2019). They have been reported to oxidise arsenic and chromium and immobilise arsenic, copper, cobalt, cadmium, nickel, zinc, and lead through complexation and isomorphic substitution for manganese (Wang et al. 2009; Liao et al. 2013; Bolan et al. 2014) (Table 3).

**Table 3:** Mn(II) oxidation by Pseudomonadota

Order	Genus/species	Isolation site	Initial Mn concentration [mM]	Mn removal efficiency	Suggested oxidation mechanism	Additional detoxification mechanisms	References
Hyphomicrobiales	*Ochrobactrum* sp.	River	10	99.1%/48 h		Precipitation	Nguyen et al. (2020)
Burkholderiales	*Achromobacter* sp.	Acid mine drainage	0.55–4.37	40–80%/120 h		Adsorption	Mao et al. (2022)
Lysobacterales	*Stenotrophomonas* sp.	Water (well)	0.109	28%/168 h		Biofilm formation	Calderón-Tovar et al. (2020)
	*Stenotrophomonas* sp.	Water (well)	0.109	20%/168 h		Biofilm formation	Calderón-Tovar et al. (2020)
	*Stenotrophomonas* sp.	Water (manganese mine)	0.91	70.9%/168 h	Nonenzymatic oxidation; pH rise		Barboza et al. (2015)
	*Stenotrophomonas* sp.	Water (manganese mine)	0.91	66.4%/168 h	Nonenzymatic oxidation; pH rise		Barboza et al. (2015)
Vibrionales	*Vibrio* sp.	Coastal seawater	1	45%/168 h			Yang et al. (2023)
Enterobacterales	*Serratia marcescens*	Wastewater	0.64	64.25%/8 h			Queiroz et al. (2018)
	*Serratia marcescens*	Wastewater	0.64	64.25%/8 h			Queiroz et al. (2018)
	*Citrobacter freundii*	Biological activated carbon (BAC) filter column	0.96	76.2%/ 96 h (55.5%-oxidation)	Multicopper oxidase genes	Adsorption	Tang et al. (2014)
Pseudomonadales	*Pseudomonas* sp.	Estuary	0.1	53.2%/96 h	Multicopper oxidase		Wright et al. 2018
	*P. hunanensis*	Estuary	0.1	91.1%/96 h	Multicopper oxidase		Wright et al. (2018)
	*Pseudomonas* sp.	Metallurgical wastewater	1.82	100%/48 h	Multicopper oxidase		Kitjanukit et al. (2019)
Alteromonadales	*Marinobacter* sp.	Seawater	10	100%/240 h (76.4%—oxidation)		Adsorption	Liao et al. (2013)
Aeromonadales	*Aeromonas* hydrophila	Mn- contaminated stream sediments	10–20	80–90%/ 144 h (30–45.6% oxidation)	pH rise	Adsorption, accumulation, precipitation	Zhang et al. (2019)
	*Aeromonas* sp.	Water (well)	0.109	30%/168 h			Calderón-Tovar et al. (2020)
	*Aeromonas* sp.	Water (well)	0.109	36%/168 h			Calderón-Tovar et al. (2020)
	*Aeromonas* sp.	Water (well)	0.109	45%/168 h		Biofilm formation	Calderón-Tovar et al. (2020)
Moraxellales	*Acinetobacter* sp.	Activated sludge	0.91–5.46	96–98%/144 h			An et al. (2021)
	*Acinetobacter* sp.	Seawater	1	70%/144 h	Enzymatic pathway		Hosseinkhani and Emtiazi (2011)
	*Acinetobacter* sp.	As, Mn contaminated soil	0.09–0.46	60%/10 h		Adsorption, coprecipitation of As	Singh et al. (2016)

### Arsenic

Stable inorganic arsenic compounds are primarily trivalent arsenite and pentavalent arsenate (Bahar et al. 2016). Under oxidising conditions, As(V) prevails and usually forms oxyanions of arsenic acid. Under anoxic or reducing conditions, trivalent arsenic species also occur, mainly as non-ionised arsenious acid (Katsoyiannis and Zouboulis 2004; Ito et al. 2012). Both arsenic forms are toxic to animals, plants, and microorganisms. Pentavalent ions are easily transported through biological membranes (Ghosh et al. 2015). They bind to sulfhydryl groups of proteins, impairing the functions of enzymes and hormones (Banerjee et al. 2011; Bahar et al. 2016; Tripti et al. 2017). As(III), on the other hand, can enter the cells through phosphate transporters and disrupt phosphorylation reactions (Banerjee et al. 2011; Ghosh et al. 2015).

Similarly to manganese, arsenic is less soluble and less toxic when it occurs in the oxidised form, mostly due to its higher affinity to ferric (hydr)oxides and aluminium hydrous oxides (Casiot et al. 2003; Das et al. 2016). These compounds strongly adsorb As(V), making it much less mobile and less available to plants (Ghosh et al. 2015; Das et al. 2016). Moreover, co-precipitation of arsenate with biogenic iron oxides has been also reported (Katsoyiannis and Zouboulis 2006). As a result, pentavalent form of arsenic is estimated to be 100 times less harmful to organisms than arsenite (Batool et al. 2017; Tripti et al. 2017).

Microorganisms use diverse pathways to counteract arsenic toxicity, including oxidation, reduction, and methylation (Batool et al. 2017; Sher and Rehman 2019). Since arsenate is less bioavailable, arsenic-oxidising bacteria are a group of particular importance for the immobilisation of toxic ions. These organisms transform As(III) into As(V) in order to reduce its toxicity or to acquire energy for growth (Banerjee et al. 2011; Ito et al. 2012; Bahar et al. 2016). Many arsenic-oxidising bacteria are chemolithoautotrophs, active both in aerobic and nitrate-reducing environments (Bachate et al. 2012; Bolan et al. 2014). Their ability to oxidise As(III) is associated with the presence of two operons: aio and arx, encoding two different arsenite oxidases (Lu et al. 2018). Although both oxidases are molybdoenzymes of dimethyl sulfoxide reductase family, they are only distantly related (Badilla et al. 2018; Szyttenholm et al. 2020). As(III) oxidase Aio (until 2012 largely referred to as “Aox”) is composed of two subunits: catalytic molybdopterin-containing *aio*A and Rieske subunit *aio*B (Branco et al. 2009; Badilla et al. 2018). The alternative (anaerobic) As(III) oxidase Arx also consists of two subunits—*arx*A (molybdopterin oxidoreductase), and a small iron-sulfur *arx*B, analogous to Rieske subunit *aio*B (Zargar et al. 2010; Szyttenholm et al. 2020). In Pseudomonadota the expression of *aio*AB is regulated by a three-gene cluster *aio*XSR (Badilla et al. 2018), while the *arx*AB gene cluster is accompanied by regulatory genes *arx*XSR (Hernandez-Maldonado et al. 2017). Although anaerobic As(III) oxidase has been found solely among Pseudomonadota, mostly in the classes Beta- and Gammaproteobacteria (Ospino et al. 2019), the majority of arsenite oxidasers belonging to this phylum harbour the aio operon (Table 4). Microbial-induced arsenic oxidation is much faster than abiotic processes (which can occur under the influence of manganese oxides or oxygen released from the plant roots) (Casiot et al. 2003; Katsoyiannis and Zouboulis 2004; Wang et al. 2020).

**Table 4:** As(III) oxidation by Pseudomonadota (with original gene nomenclature)

Order	Genus/species	Isolation site	Initial As concentration [mM]	As removal efficiency	Suggested oxidation mechanism	Additional detoxification mechanisms	References
Hyphomicrobiales	*Ochrobactrum tritici*	Wheat rhizosphere	5	100%/14 h	As(III) oxidase (aoxAB), c-type cytochrome (cytC), molybdopterin biosynthesis (moeA)		Branco et al. (2009)
	*Agrobacterium albertimagni*	Surface of aquatic macrophytes	0.585	ca. 88%/22 h	Arsenite oxidase		Salmassi et al. (2002)
	*A. tumefaciens*	Soil	0.075	99.9%/35 h			Macur et al. 2004
	*A. tumefaciens*		1.33	> 97%/18 h	Arsenite oxidase (aoxB)		Fan et al. (2008)
	*Bosea* sp.	As-rich groundwater	0.25	70%/144 h	aoxB		Liao et al. (2013)
	*Bosea* sp.	Sb mine slag	2	100%/24 h	As(III) oxidase and its regulating proteins(aioX, aioS, aioR, aioA, aioB)		Lu et al. (2018)
	*Sinorhizobium* sp.	Sb mine tailing area	10	87–100%/168 h			Hamamura et al. (2013)
	*Ensifer* sp.	Activated sludge	1,5	> 80%/360 h	As(III) oxidase (aioA)		Ito et al. (2012)
	*Sinorhizobium* sp.		0.67	> 95%/38 h	Arsenite oxidase (aoxB)		Fan et al. (2008)
	*Rhodopseudomonas faecalis*	Fish pond	5	96%/5 h	Enzymatic pathway		Batool et al. (2017)
Rhodobacterales	*Paracoccus* sp.	As-contaminated paddy soil	1	100%/24 h	As(III) oxidase (aioA)		Zhang et al. (2015)
Rhodospirillales	*Azospirillum* sp.		0.1	92%/8 h	As(III) oxidase (aioA) (membrane)		Bahar et al. (2016)
Burkholderiales	*Achromobacter xylosoxidans*	Soil	2–5	75–100%/10 h	As(III) oxidase		Bachate et al. (2012)
	*Achromobacter* sp.	Groundwater sediments	1.33	ca. 94%/8 h	As(III) oxidase (aoxB)		Fan et al. (2008)
	*Achromobacter* sp.	As-contaminated soil	0.8	100%/13 h	As(III) oxidase (gene clusters aoxXSR and aoxABCD)		Cai et al. (2009)
	*Alcaligenes* sp.	Mine soil	1	100%/40 h			Yoon et al. (2009)
	*Bordetella* sp.		2–5	85–100%/8 h	As(III) oxidase		Bachate et al. (2012)
	*Thiomonas* sp.	Mine tailings	3.09	75%/150 h		Fe oxidation, precipitation	Casiot et al. (2003)
	*Noviherbaspirillum* sp.	As-contaminated paddy soil	0.01	60%/48 h		Precipitation	Wu et al. (2021)
	*Variovorax paradoxus*	Soil	0.075	100%/50 h			Macur et al. (2004)
	*Acidovorax* sp.		0.67	> 95%/39 h	As(III) oxidase (aoxB)		Fan et al. (2008)
Rhodocyclales	*Zoogloea* sp.		1.33	100%/72 h			Weeger et al. (1999)
Lysobacterales	*Stenotrophomonas* sp.	Soil (Pb smelter)	0.93–6.67	100%/12 h			Bahar et al. (2012)
Pseudomonadota	*P. fluorescens*	Soil	0.075	ca. 73%/ 37 h			Macur et al. (2004)
	*Pseudomonas* sp.	As-contaminated soil	0.8	100%/12 h	As(III) oxidase (cluster arsDA-aoxAB)		Cai et al. (2009)
	*P. arsenicoxydans*	Sediments, desert	6.67	100%/48 h			Campos et al. (2010)
	*P. mendocina*	Mine tailings	0.5	100%/72 h		Siderophores	Miranda-Carrazco et al. (2018)
	*Pseudomonas* sp.	As contaminated soil	6–8	25–100%/144 h		Accumulation, absorption	He et al. (2023)
	*Pseudomonas* sp.	Mine soil	0.067	92.0%/ 3 h			Zhang et al. (2016)
	*Pseudomonas* sp.	Arsenic-rich gold mine tailings	2.67	80%/12 h			Karn and Pan (2017)
	*Pseudomonas* sp.		1	31%/24 h		Siderophores	Ghosh et al. (2015)
	*Pseudomonas* sp.		1	46%/24 h		Siderophores	Ghosh et al. (2015)
Alteromonadales	*Marinobacter santoriniensis*		2.5–5	80–100%/144 h	As(III) oxidase (aoxB)		Handley et al. (2009)
	*Marinobacter* sp.	Seawater	0.055	> 90%/144 h (> 70%–oxidation)	Mn oxides		Liao et al. (2013)
Moraxellales	*A. calcoaceticus*	As-contaminated soil	1	ca. 50%/96 h		Siderophores, sorption, accumulation	Banerjee et al. (2011)
	*A. lwoffi*	As-contaminated soil	1	97.2%/144 h		Siderophores, sorption, accumulation	Banerjee et al. (2011)

Some arsenic-oxidising bacteria exhibit additional beneficial traits such as the ability to solubilize phosphates or to release plant-growth promoting compounds. These include indole-3-acetic acid (IAA)—a plant hormone responsible for cell division and elongation, 1-aminocyclopropane-1-carboxylate (ACC) deaminase, which limits the biosynthesis of stress hormone ethylene, and siderophores—Fe(III)-binding compounds, which increase the bioavailability of iron (Das et al. 2016; Wang et al. 2020). It has been observed that species such as *Vigna mungo* or *Oryza sativa* (crop plant particularly exposed to arsenic toxicity) not only accumulated less arsenic in the tissues, but also grew faster, reached higher biomass, and produced more photosynthetic pigments after inoculation with arsenite-oxidising bacteria (Das et al. 2016; Batool et al. 2017; Wang et al. 2020). In addition, microorganisms that produce more siderophores exhibit higher resistance to arsenic toxicity and greater oxidising capabilities (Ghosh et al. 2015; Nookongbut et al. 2018). This phenomenon can be explained by better iron nutrition—necessary for the activation of arsenite oxidase—and enhancement of iron plaque formation, which helps retain arsenic outside the root (Ghosh et al. 2015, Lakshmanan et al. 2015).

## The contribution of Pseudomonadota to biotransformation

**Fig. 3:**
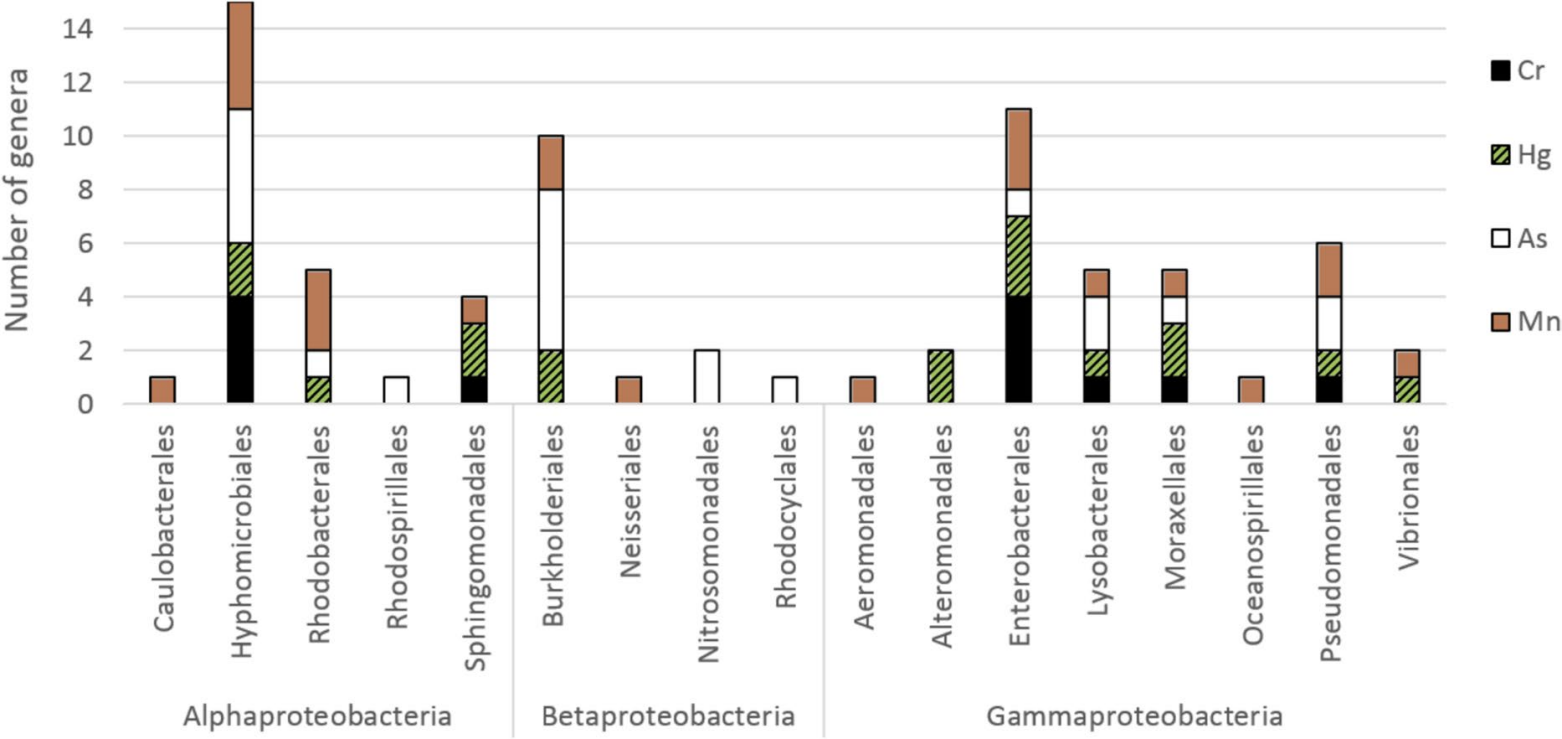
Number of genera from three orders in the phylum Pseudomonadota, reported to be involved in redox detoxification of Cr, Hg, As, and Mn (list of references in Online Resource S1)

The two most abundant classes of Pseudomonadota, Alpha- and Gammaproteobacteria, are involved in redox transformations of all four elements. This confirms the ability of their members to perform both oxidation and reduction processes (Fig. 3). Conversely, Betaproteobacteria participate almost exclusively in the detoxification of arsenic and manganese, which is due to their well-known oxidative abilities. Such genera as *Gallionella* (Nitrosomonadales) or *Leptothrix* (Burkholderiales) are iron-oxidising bacteria (Emerson et al. 2010), while *Thiobacillus* (Nitrosomonadales) and *Thiomonas* (Burkholderiales) contribute to sulphur oxidation (Ranadev et al. 2023). Moreover, almost all ammonia oxidising bacteria in the land environment belong to the class Betaproteobacteria (Norton 2014). Nevertheless, several marine *Alcaligenes faecalis* isolates have been shown to reduce mercury (De et al. 2008).

Among Alphaproteobacteria, Hyphomicrobiales (Rhizobiales) are particularly rich in strains that detoxify trace elements by modifying their oxidation state. This order contains plant-associated diazotrophs and methanotrophs, but also free-living phototrophic purple bacteria, which are able to transform chrome, arsenic, manganese, and mercury into less toxic form (Dhar et al. 2023). The best studied example of such an organism is *Ochrobactrum* sp. A number of strains, belonging i.a. to *O. anhropi*, *O. tritici*, *O. chromiisoli*, turned out to be useful in the process of biotransformation (Branco et al. 2009; Chang et al. 2024; Yang et al. 2024). Moreover, some *Ochrobactrum* strains showed the ability to produce biosurfactants and siderophores (Chakraborty and Das 2014; Malaviya and Singh 2016) and absorb trace elements such as arsenic, chromium, copper, and cadmium (Aksornchu et al. 2007; François et al. 2012; Aryal and Liakopoulou-Kyriakides 2015; Caracciolo and Terenzi 2021).

There are few studies concerning the involvement of Hyphomicrobiales in mercury volatilization; nevertheless, such genera as *Afifella* spp. and *Xanthobacter* spp. have been shown to transform Hg(II) into less harmful Hg(0) form (Mukkata et al. 2015; Petrus et al. 2015). Moreover, Zhu et al. (2021) observed a significant increase in the abundance of families Rhizobiaceae and Nitrobacteraceae in soils containing high mercury concentrations. This result led the authors to suggest that *Rhizobium* spp. could be treated as a biomarker of mercury-polluted sites (Zhu et al. 2021).

Gammaproteobacteria are not only very efficient, but also versatile biotransforming microorganisms. Bacteria belonging to four orders (Enterobacterales, Lysobacterales, Moraxellales, and Pseudomonadales) are involved in redox detoxification processes of all four trace elements covered in our review. In this context, three genera of ubiquitous and environmentally important bacteria deserve special attention. These are: *Stenotrophomonas* (Lysobacterales), *Acinetobacter* (Moraxellales), and *Pseudomonas* (Pseudomonadales).

Members of the genus *Stenotrophomonas* are usually plant-associated organisms, isolated both from the rhizosphere and internal plant tissues (Ryan et al. 2009). They play an important ecological role, participating in the nitrogen and sulphur cycles, as well as producing extracellular enzymes (Ryan et al. 2009). Many *Stenotrophomonas* strains are resistant to heavy metal toxicity and are good candidates for bioremediation agents (Ryan et al. 2009). Plant growth-promoting *S. rhizophila* isolated from oilseed rape rhizosphere has been shown to completely reduce 50 mg/l hexavalent chromium in 28 h (Gao et al. 2020), while *S. maltophilia* originating from chromate-contaminated wastewater, was able to reduce 92% of metal ions with the initial concentration of 500 mg/l (Baldiris et al. 2018). Furthermore, a strain of *S. maltophilia* isolated by Gunasundari and Muthukumar (2013) from polluted soil has been shown to simultaneously reduce chromate and degrade phenol. Barboza et al. (2015) demonstrated that *Stenotrophomonas* isolates originating from a manganese mine can oxidise Mn(II) by a nonenzymatic pathway involving pH rise. Bahar et al. (2016), on the other hand, reported a complete oxidation of 500 μM arsenite by a strain closely related to *S. panacihumi*.

Some *Stenotrophomonas* strains possess additional properties—other than redox abilities—that make them useful as bioremediation agents. In particular, they are able to produce extracellular polysaccharides and form biofilm, which facilitates the processes of biosorption and bioaccumulation of toxic ions (Ge and Ge 2016; Calderón-Tovar et al. 2020). Song et al. (2022) observed that the contribution of different mechanisms leading to manganese removal changes depending on the concentration of Mn(II); higher values (50 mM) promoted oxidation, while lower ones (10–40 mM) induced MnCO_3_ precipitation.

Another gammaproteobacterial genus displaying great potential in redox transformation of trace elements is *Acinetobacter*. These widespread, strictly aerobic microorganisms are not only adapted to a broad range of habitats, differing in temperature and humidity, but they are also resistant to multiple metal ions and antibiotics (Doughari et al. 2011; Jung and Park 2015; Sevak et al. 2023). Their metabolic capabilities can be used in bioremediation of both heavy metal- and hydrocarbon- contaminated sites (Dahal et al. 2023).

A number of *Acinetobacter* species have been reported to reduce hexavalent chromium. These include *A. baumannii*, *A. calcoaceticus*, *A. haemolyticus*, *A. indicus*, *A. junii*, and *A. radioresistens*, among others (Samantaray and Mishra 2012; Ahmad et al. 2013; Sathishkumar et al. 2017; Ram Talib et al. 2019; Hu et al. 2021; Sevak et al. 2023). Some of them employ other mechanisms to detoxify metal ions, such as intracellular accumulation, efflux or adsorption at the cell wall (Essahale et al. 2012; Bhattacharya and Gupta 2013; Ahmad et al. 2013; Sevak et al. 2023). Manganese- and arsenic-oxidising strains have also been shown to sequester toxic ions by the process of adsorption and precipitation (Singh et al. 2016; An et al. 2021; Zhao et al. 2023).

The oxidation of Mn(II) by *Acinetobacter* sp. can be related to other biochemical processes, most notably those involved in the nitrogen cycle (Wang et al. 2022). An et al. (2021) reported that a nitrifying *Acinetobacter* strain was able to simultaneously remove 99.05% manganese and 64.23% of NH^4+^-N. Su et al. (2016), on the other hand, noted that manganese oxidation performed by a bacterium isolated from an oligotrophic lake was coupled with anaerobic denitrification.

Most biotransforming *Acinetobacter* strains have been isolated from polluted environments, such as mercury and chromite mines, tannery and dyehouse effluents, contaminated soils and waters (Essahale et al. 2012; Pulimi 2012; Campos-Guillén et al. 2014; Hu et al. 2021). Some of them, however, originate from agricultural soil or inhabit plant tissues. These isolates often show plant growth-promoting traits, which makes them even more beneficial for the remediation of polluted sites (Banerjee et al. 2011; Mello et al. 2020; Dutta et al. 2024).

*Pseudomonas* is the most species-rich genus of gram-negative bacteria (Lalucat et al. 2022). Its wide distribution and metabolic diversity allows multiple applications in agriculture and bioremediation (Misra et al. 2022; Fakhar et al. 2022). Almost one third of biotransforming Pseudomonadota strains (and over a half in the case of mercury) belong to this genus. A number of *Pseudomonas* species, including *P. aeruginosa*, *P. fluorescens*, *P. pseudoalcaligenes*, *P. putida*, and *P. umsongensis*, are able to volatilize Hg(II) (Belzile et al. 2006; De et al. 2008; Zhang et al. 2012; Yao et al. 2020; Biełło et al. 2023). Manganese-oxidising bacteria of the genus *Pseudomonas* are mostly water isolates belonging to such species as *P. guangdongensis*, *P. hunanensis*, *P. resinovorans*, and *P. taiwanensis* (Wright et al. 2018; Piazza et al. 2019; Liu et al. 2022); arsenite-oxidising strains (e.g. *P. arsenicoxydans*, *P. mendocina*), conversely, usually originate from arsenic-contaminated soils (Campos et al. 2010; Miranda-Carrazco et al. 2018; Yang et al. 2023; He et al. 2023).

Some biotransforming *Pseudomonas* strains have been shown to produce siderophores, biosurfactants, and promote plant growth (Zawadzka et al. 2007; Ozturk et al. 2012; Miranda-Carrazco et al. 2018; Shi et al. 2023). In addition, such organisms as mercury-volatilising *P. pseudoalcaligenes* and arsenite-oxidising *P. mendocina* are cyanotrophs, able to use cyanide as the sole nitrogen source (Miranda-Carrazco et al. 2018; Biełło et al. 2023).

## Risks and challenges

There are still a lot of questions concerning the applicability of microorganisms for the redox-based remediation of heavy metal-contaminated soils and waters. First of all, redox reactions require both an electron acceptor and an electron donor. In the most favourable cases the reduction of one trace element is coupled with the oxidation of another one, such as simultaneous chromate reduction and arsenic oxidation performed by *Ensifer adherens* (Li et al. 2023). More often the biotransformation is associated with basic metabolic processes, including nitrogen and sulphur cycles. However, it is possible that the redox process leading to the detoxification of one heavy metal will result in the transformation of another one into its more hazardous form. For example, several studies showed that the formation of biogenic manganese oxides can lead to the oxidation of Cr(III) (Murray et al. 2005; Wu et al. 2005; He et al. 2009). Interestingly, trivalent chromium, generally considered less harmful than chromate, proved to be more toxic to the *P. putida* isolate tested by Murray et al. (2005). The indirect oxidation of Cr(III) was then a means to mitigate oxidative stress.

Many genera in the phylum Pseudomonadota show both reductive and oxidative abilities towards various ions. Such is the case for *Serratia* spp. which can reduce chromate and oxidise manganese (González et al. 2014; Queiroz et al. 2018), and *Alcaligenes* spp. which volatilise mercury and oxidise As(III) (De et al. 2008; Yoon et al. 2009). Sometimes a single bacterial genus or species can induce the biotransformation of an element “in both directions”. For example, some genera whose members are known to oxidise manganese (e.g. *Pseudomonas*, *Acinetobacter*) include efficient reducers of Mn(IV), too (Gounot 1994). Similarly, different *E. coli* strains have been shown to transform elemental mercury to Hg(II) and to volatilise the divalent ion into its elemental form (Smith et al. 1998; Dash et al. 2017). Macur et al. (2004) isolated two *Agrobacterium tumefaciens* strains with almost identical rDNA sequences, each of which performed an opposite redox transformation of arsenic ions. Furthermore, Su et al. (2016) described a *P. stutzeri* strain able to reduce or oxidise arsenic, depending on the availability of oxygen. Selecting a bacterial isolate which would show the desired properties in a range of conditions could be therefore a challenge.

Importantly, some members of the Gammaproteobacteria class reported as effective reducing or oxidizing agents are human pathogens. This holds in particular for Enterobacterales, but also for *Acinetobacter* spp., *Stenotrophomonas* spp., and *Pseudomonas* spp. *Acinetobacter* strains are responsible for skin, blood, and urinary tract infections, cholangitis, peritonitis, endocariditis, respiratory diseases, meningitis, and ventriculitis (Doughari et al. 2011). Such species as *A. baumannii*, *A. calcoaceticus*, *A. lwoffii*, and *A. radioresistens* are known to cause pneumonia, and cases of human infections with *A. junii* and *A. haemolyticus* were also reported (Dahal et al. 2023). Another common cause of nosocomial diseases are *Stenotrophomonas* species, especially those classified as “*S. maltophilia* complex”, including i.a. *S. maltophilia* and *S. pavanii* (Svensson-Stadler et al. 2012; Li et al. 2024). These multidrug-resistant opportunistic pathogens are associated with blood, skin, eye, and respiratory tract infections (Li et al. 2024). The best studied bacterial pathogen of the genus *Pseudomonas* is *P. aeruginosa*, one of the most common causes of bacteremia, urinary tract infections, and pneumonia in hospitals, and it is probably responsible for over 7% of all healthcare-associated infections (Reynolds and Kollef 2021). However, other *Pseudomonas* species can also become opportunistic human pathogens; these include *P. fluorescens*, *P. mendocina*, *P. putid*a, and *P. stutzeri* (Gani et al. 2019; Alwazzeh et al. 2020; Singh et al. 2021). Moreover, some microorganisms belonging to this genus are pathogenic to insects (e.g. *P. entomophila*, *P. taiwanensis*), which make them a risky choice for environmental applications (Chen et al. 2014; Dieppois et al. 2015).

However, it should be noted that, in reality, the risk of infection may vary significantly between aquatic and soil environments, as well as between in situ systems, where contact with pathogens can be more direct and difficult to control and ex situ systems, where bioremediation processes can be conducted in more contained and sterile conditions. For example, aquatic environments pose a particularly high risk of pathogen transmission due to the ease with which bacteria spread and contaminate drinking water sources or recreational waters. Even low levels of inoculation can lead to epidemiological consequences (Some et al. 2021; Kristanti et al. 2022). On the other hand, soils are generally considered less risky, but many pathogens can persist in them for long periods and indirectly infect humans, for example through food (Santamaría and and Toranzos 2003; Bultman et al. 2012; DeFlorio et al. 2021). The risk is especially high near agricultural or recreational areas. Thus, in situ systems are difficult to control, increasing the likelihood of microbial spread and contact with humans or animals. Conversely, ex situ systems provide greater safety by allowing controlled conditions and containment of pathogens. Closed bioreactors, filtration, and pasteurization can be implemented to minimize residual risk (Perez-Vazquez et al. 2024).

It is also worth noting that if a pathogen shows high detoxification efficiency and is considered for in situ application, the associated risk could be mitigated by eliminating its pathogenicity. This can be achieved, for example, by silencing virulence-related genes using CRISPRi system (Zhang et al. 2021). However, genetically modified bacteria (GMB), including those engineered for increased expression of metal-detoxifying genes, may pose several ecological risks. One major concern is horizontal gene transfer (HGT), where GMB could transfer recombinant genes to native microbial populations, leading to the unintended spread of modified traits (Alishah Aratboni et al. 2019). This process occurs spontaneously through transduction, conjugation, or other mechanisms, with interspecies transfer documented across various ecological conditions (Villegas-Torres et al. 2011). While HGT is a natural driver of bacterial genome innovation (Sobecky and Coombs 2009), engineered systems often fail to account for field conditions and complex ecological interactions (Singh et al. 2011). Current mitigation strategies include suicide gene mechanisms and environmental containment (Zuo et al. 2015), though these may not fully prevent genetic leakage or ecological disruption.

Moreover, genetically modified bacteria and non-native bacterial strains may disrupt ecological balance by competing with native microorganisms for resources or by producing secondary metabolites, potentially altering the microbial communities (Manfredini et al. 2021; Vickram et al. 2024; Dobrzyński et al. 2025). Consequently, future research in this field should investigate the impact of both GEMs and natural isolates on native microbial communities, including taxonomic structure and the abundance of key genes involved in ecological processes. Several studies have already been published on the effects of beneficial bacteria belonging to the phylum Pseudomonadota on soil microbiota (Yin et al. 2013; Płociniczak et al. 2020; Manfredini et al. 2021; Zhang et al. 2023; Miao et al. 2023; Dobrzyński et al. 2024a, b). Importantly, in the near future, advances in sequencing technologies and bioinformatics tools will enable more accurate risk assessment of bacteria used in bioremediation. The application of metagenome-assembled genomes (MAGs) will soon allow precise evaluation of how introduced bacteria affect functional diversity; the key advantage of this approach will be the ability to assign specific environmental genes to particular taxa (Zhang et al. 2024; Wang et al. 2025). In conclusion, these unintended consequences underscore the importance of thorough risk assessments before releasing genetically modified or even native strains into the environment (Alishah Aratboni et al. 2019).

## Conclusions

Pseudomonadota are abundant and diverse, and they have a great potential in heavy metal detoxification. Two most numerous classes, Alpha- and Gammaproteobacteria, are efficient Mn(II) and As(III) oxidisers, as well as Hg(II) and Cr(VI) reducers. However, the use of biotransforming bacteria in the remediation poses a number of risks, including the reversibility of redox reactions and generation of toxic by-products. For this reason, such processes should be carried out under well researched and strictly controlled conditions.

Another concern is a potential pathogenicity of many bacterial species. This problem can be addressed by selecting noninfectious strains or using genetic engineering to silence virulence-related genes. This, however, raises another questions about the possible release of genetically modified bacteria to the environment.

Considering all this, the alphaproteobacterial order Hyphomicrobiales (including *Ochrobactrum* spp., but also biotransforming bacteria of the genera *Ensifer*, *Rhodopseudomonas*, *Hyphomicrobium*, and *Pedomicrobium*) seem particularly promising in terms of the bioremediation of contaminated sites. The activity of these non-pathogenic, environmentally important microorganisms is worth further studies to fully understand their potential. In particular, given their effectiveness in Cr(VI) reduction and As(III) oxidation, more research is needed to verify their role in Mn(II) and Hg(II) transformation.

## Supplementary Information

Below is the link to the electronic supplementary material.

Supplementary file1 (PDF 481 KB)

## Data Availability

No datasets were generated or analysed during the current study.

## References

[CR1] Ahemad M (2014) Bacterial mechanisms for Cr(VI) resistance and reduction: an overview and recent advances. Folia Microbiol 59:321–332. 10.1007/s12223-014-0304-8 24470188 10.1007/s12223-014-0304-8

[CR2] Ahmad WA, Wan Ahmad WH, Karim NA, Santhana Raj AS, Zakaria ZA (2013) Cr(VI) reduction in naturally rich growth medium and sugarcane bagasse by *Acinetobacter haemolyticus*. Int Biodeterior Biodegrad 85:571–576. 10.1016/j.ibiod.2013.01.008

[CR209] Aksornchu P, Prasertsan P, Sobhon V (2007) Isolation of arsenic-tolerant bacteria from arsenic-contaminated soil. Songklanakarin J Sci Technol 30:95–102

[CR3] Alam MZ, Ahmad S (2012) Toxic chromate reduction by resistant and sensitive bacteria isolated from tannery effluent contaminated soil. Ann Microbiol 62:113–121. 10.1007/s13213-011-0235-4

[CR4] Alam MZ, Malik A (2008) Chromate resistance, transport and bioreduction by *Exiguobacterium* sp. ZM-2 isolated from agricultural soil irrigated with tannery effluent. J Basic Microbiol 48:416–420. 10.1002/jobm.200800046 18759228 10.1002/jobm.200800046

[CR5] Alishah Aratboni H, Rafiei N, Garcia-Granados R, Alemzadeh A, Morones-Ramírez JR (2019) Biomass and lipid induction strategies in microalgae for biofuel production and other applications. Microb Cell Fact 18:1–17. 10.1186/s12934-019-1228-4 31638987 10.1186/s12934-019-1228-4 PMC6805540

[CR6] Alwazzeh MJ, Alkuwaiti FA, Alqasim M, Alwarthan S, El-ghoneimy Y (2020) Infective endocarditis caused by *Pseudomonas stutzeri*: a case report and literature review. Infect Dis Rep 12:105–109. 10.3390/idr12030020 33276629 10.3390/idr12030020 PMC7768374

[CR7] An Q, Jin L, Deng S, Li Z, Zhang C (2021) Removal of Mn(II) by a nitrifying bacterium Acinetobacter sp. AL-6: efficiency and mechanisms. Environ Sci Pollut Res 28(24):31218–31229. 10.1007/s11356-021-12764-6 10.1007/s11356-021-12764-6 33599926

[CR8] Andeer PF, Learman DR, McIlvin M, Dunn JA, Hansel CM (2015) Extracellular haem peroxidases mediate Mn(II) oxidation in a marine *Roseobacter* bacterium via superoxide production. Environ Microbiol 17:3925–3936. 10.1111/1462-2920.12893 25923595 10.1111/1462-2920.12893

[CR9] Aryal M, Liakopoulou-Kyriakides M (2015) Bioremoval of heavy metals by bacterial biomass. Environ Monit Assess 187:4173. 10.1007/s10661-014-4173-z 25471624 10.1007/s10661-014-4173-z

[CR10] Bachate SP, Khapare RM, Kodam KM (2012) Oxidation of arsenite by two β-proteobacteria isolated from soil. Appl Microbiol Biotechnol 93:2135–2145. 10.1007/s00253-011-3606-7 21983709 10.1007/s00253-011-3606-7

[CR11] Badilla C, Osborne TH, Cole A, Watson C, Djordjevic S, Santini JM (2018) A new family of periplasmic-binding proteins that sense arsenic oxyanions. Sci Rep 8:6282. 10.1038/s41598-018-24591-w 29674678 10.1038/s41598-018-24591-w PMC5908839

[CR12] Bae W, Wu CH, Kostal J, Mulchandani A, Chen W (2003) Enhanced mercury biosorption by bacterial cells with surface-displayed MerR. Appl Environ Microbiol 69:3176–3180. 10.1128/AEM.69.6.3176-3180.2003 12788714 10.1128/AEM.69.6.3176-3180.2003 PMC161548

[CR13] Bahar MdM, Megharaj M, Naidu R (2012) Arsenic bioremediation potential of a new arsenite-oxidizing bacterium *Stenotrophomonas* sp. MM-7 isolated from soil. Biodegradation 23:803–812. 10.1007/s10532-012-9567-4 22760225 10.1007/s10532-012-9567-4

[CR14] Bahar MM, Megharaj M, Naidu R (2016) Oxidation of arsenite to arsenate in growth medium and groundwater using a novel arsenite-oxidizing diazotrophic bacterium isolated from soil. Int Biodeterior Biodegrad 106:178–182. 10.1016/j.ibiod.2015.10.019

[CR15] Baldi F, Parati F, Semplici F, Tandoi V (1993) Biological removal of inorganic Hg(II) as gaseous elemental Hg(0) by continuous culture of a Hg-resistant *Pseudomonas putida* strain FB-1. World J Microbiol Biotechnol 9:275–279. 10.1007/BF00327854 24419964 10.1007/BF00327854

[CR16] Baldiris R, Acosta-Tapia N, Montes A, Hernández J, Vivas-Reyes R (2018) Reduction of hexavalent chromium and detection of chromate reductase (ChrR) in *Stenotrophomonas maltophilia*. Molecules 23:406. 10.3390/molecules23020406 29438314 10.3390/molecules23020406 PMC6017488

[CR17] Banerjee S, Datta S, Chattyopadhyay D, Sarkar P (2011) Arsenic accumulating and transforming bacteria isolated from contaminated soil for potential use in bioremediation. J Environ Sci Health A 46:1736–1747. 10.1080/10934529.2011.623995 10.1080/10934529.2011.623995 22175878

[CR18] Banerjee S, Misra A, Chaudhury S, Dam B (2019) A *Bacillus* strain TCL isolated from Jharia coalmine with remarkable stress responses, chromium reduction capability and bioremediation potential. J Hazard Mater 367:215–223. 10.1016/j.jhazmat.2018.12.038 30594722 10.1016/j.jhazmat.2018.12.038

[CR19] Barboza NR, Amorim SS, Santos PA, Reis FD, Cordeiro MM, Guerra-Sá R, Albis Leão V (2015) Indirect manganese removal by *Stenotrophomonas* sp. and *Lysinibacillus* sp. isolated from brazilian mine water. BioMed Res Int 2015:1–14. 10.1155/2015/925972 10.1155/2015/925972 PMC4678070 26697496

[CR20] Barra Caracciolo A, Terenzi V (2021) Rhizosphere microbial communities and heavy metals. Microorganisms 9:1462. 10.3390/microorganisms9071462 34361898 10.3390/microorganisms9071462 PMC8307176

[CR21] Batool R (2012) Hexavalent chromium reduction by bacteria from tannery effluent. J Microbiol Biotechnol 22:547–554. 10.4014/jmb.1108.08029 22534304 10.4014/jmb.1108.08029

[CR22] Batool K, Tuz Zahra F, Rehman Y (2017) Arsenic-redox transformation and plant growth promotion by purple nonsulfur bacteria *Rhodopseudomonas palustris* CS2 and *Rhodopseudomonas faecalis* SS5. Biomed Res Int 2017:1–8. 10.1155/2017/6250327 10.1155/2017/6250327 PMC5366193 28386559

[CR23] Belzile N, Wu GJ, Chen Y-W, Appanna VD (2006) Detoxification of selenite and mercury by reduction and mutual protection in the assimilation of both elements by *Pseudomonas fluorescens*. Sci Total Environ 367:704–714. 10.1016/j.scitotenv.2006.03.008 16626785 10.1016/j.scitotenv.2006.03.008

[CR24] Bhattacharya A, Gupta A (2013) Evaluation of *Acinetobacter* sp. B9 for Cr (VI) resistance and detoxification with potential application in bioremediation of heavy-metals-rich industrial wastewater. Environ Sci Pollut Res 20:6628–6637. 10.1007/s11356-013-1728-4 10.1007/s11356-013-1728-4 23619927

[CR25] Biełło KA, Olaya-Abril A, Cabello P, Rodríguez-Caballero G, Sáez LP, Moreno-Vivián C, Luque-Almagro VM, Roldán MD (2023) Quantitative proteomic analysis of cyanide and mercury detoxification by *Pseudomonas pseudoalcaligenes* CECT 5344. Microbiol Spectr 11:e00553-23. 10.1128/spectrum.00553-23 37432117 10.1128/spectrum.00553-23 PMC10433974

[CR26] Binish MB, Shini S, Sinha RK, Krishnan KP, Mohan M (2021) Mercuric reductase gene (merA) activity in a mercury tolerant sulphate reducing bacterium isolated from the Kongsfjorden. Arctic. Polar Science 30:100745. 10.1016/j.polar.2021.100745

[CR27] Bolan N, Kunhikrishnan A, Thangarajan R, Kumpiene J, Park J, Makino T, Kirkham MB, Scheckel K (2014) Remediation of heavy metal(loid)s contaminated soils—to mobilize or to immobilize? J Hazard Mater 266:141–166. 10.1016/j.jhazmat.2013.12.018 24394669 10.1016/j.jhazmat.2013.12.018

[CR28] Bopp LH, Ehrlich HL (1988) Chromate resistance and reduction in *Pseudomonas fluorescens* strain LB300. Arch Microbiol 150:426–431. 10.1007/BF00422281

[CR29] Branco R, Francisco R, Chung AP, Morais PV (2009) Identification of an *aox* system that requires cytochrome *c* in the highly arsenic-resistant bacterium *Ochrobactrum tritici* SCII24. Appl Environ Microbiol 75:5141–5147. 10.1128/AEM.02798-08 19525272 10.1128/AEM.02798-08 PMC2725503

[CR30] Briffa J, Sinagra E, Blundell R (2020) Heavy metal pollution in the environment and their toxicological effects on humans. Heliyon 6:e04691. 10.1016/j.heliyon.2020.e04691 32964150 10.1016/j.heliyon.2020.e04691 PMC7490536

[CR31] Bultman MW, Fisher FS, Pappagianis D (2012) The ecology of soil-borne human pathogens. In Essentials of medical geology: revised edition. Dordrecht: Springer Netherlands, pp 477–504. 10.1007/978-94-007-4375-5_20

[CR32] Cai L, Rensing C, Li X, Wang G (2009) Novel gene clusters involved in arsenite oxidation and resistance in two arsenite oxidizers: *Achromobacter* sp. SY8 and *Pseudomonas* sp. TS44. Appl Microbiol Biotechnol 83:715–725. 10.1007/s00253-009-1929-4 19283378 10.1007/s00253-009-1929-4

[CR33] Cai Y, Yang K, Qiu C, Bi Y, Tian B, Bi X (2023) A review of manganese-oxidizing bacteria (MnOB): applications, future concerns, and challenges. Int J Environ Res Public Health 20:1272. 10.3390/ijerph20021272 36674036 10.3390/ijerph20021272 PMC9859543

[CR34] Calderón-Tovar IL, Rietveld LC, Araya-Obando JA, Quesada-González A, Caballero-Chavarría A, Romero-Esquivel LG (2020) Autochthonous tropical groundwater bacteria involved in manganese(II) oxidation and removal. Environ Sci- Water Res Technol 6:3132–3141. 10.1039/D0EW00704H

[CR35] Campos VL, Valenzuela C, Yarza P, Kämpfer P, Vidal R, Zaror C, Mondaca M-A, Lopez-Lopez A, Rosselló-Móra R (2010) *Pseudomonas arsenicoxydans* sp nov., an arsenite-oxidizing strain isolated from the Atacama desert. Syst Appl Microbiol 33:193–197. 10.1016/j.syapm.2010.02.007 20409659 10.1016/j.syapm.2010.02.007

[CR36] Campos-Guillén J, Caballero Pérez J, Cruz Medina JA, Molina Vera C, Salas Rosas LM, Limpens Gutiérrez C, García Salinas I, Hernández Ramírez MR, Soto Alonso G, Cruz Hernández A, Saldaña Gutiérrez C, Romero Gómez S, Pastrana Martínez X, Álvarez Hidalgo E, Gosar M, Dizdarevič T (2014) Draft genome sequence of the mercury-resistant bacterium *Acinetobacter idrijaensis* strain MII, isolated from a mine-impacted area, Idrija, Slovenia. Genome Announc 2:e01177-14. 10.1128/genomeA.01177-14 25395645 10.1128/genomeA.01177-14 PMC4241671

[CR37] Casiot C, Morin G, Juillot F, Bruneel O, Personné J-C, Leblanc M, Duquesne K, Bonnefoy V, Elbaz-Poulichet F (2003) Bacterial immobilization and oxidation of arsenic in acid mine drainage (Carnoulès creek, France). Water Res 37:2929–2936. 10.1016/S0043-1354(03)00080-0 12767295 10.1016/S0043-1354(03)00080-0

[CR208] Chakraborty J, Das S (2014) Biosurfactant-based bioremediation of toxic metals. In: Microbial Biodegradation and Bioremediation. Elsevier, pp 167–201

[CR38] Chang JS, Law WS (1998) Development of microbial mercury detoxification processes using mercury-hyperresistant strain of *Pseudomonas aeruginosa* PU21. Biotechnol Bioeng 57:462–470 10099223

[CR207] Chang JS, Kim HJ, Lee JH (2024) Detoxification of ars genotypes by arsenite–oxidizing bacteria through arsenic biotransformation. Environ Geochem Health 46:470. 10.1007/s10653-024-02251-5 10.1007/s10653-024-02251-5 39382695

[CR39] Chen W-J, Hsieh F-C, Hsu F-C, Tasy Y-F, Liu J-R, Shih M-C (2014) Characterization of an insecticidal toxin and pathogenicity of *Pseudomonas taiwanensis* against insects. PLoS Pathog 10:e1004288. 10.1371/journal.ppat.1004288 25144637 10.1371/journal.ppat.1004288 PMC4140846

[CR40] Chen J, Dong J, Shen S, Mei J, Chang J (2019) Isolation of the Hg(II)-volatilizing *Bacillus* sp. strain DC-B2 and its potential to remediate Hg(II)-contaminated soils. J Chem Tech Biotech 94:1433–1440. 10.1002/jctb.5905

[CR41] Cheung KH, Lai HY, Gu JD (2006) Membrane-associated hexavalent chromium reductase of *Bacillus megaterium* TKW3 with induced expression. J Microbiol Biotechnol 16:855–862

[CR42] Chien M, Nakahata R, Ono T, Miyauchi K, Endo G (2012) Mercury removal and recovery by immobilized *Bacillus megaterium* MB1. Front Chem Sci Eng 6:192–197. 10.1007/s11705-012-1284-3

[CR43] Dahal U, Paul K, Gupta S (2023) The multifaceted genus *Acinetobacter*: from infection to bioremediation. J Appl Microbiol 134:lxad145. 10.1093/jambio/lxad145 37442632 10.1093/jambio/lxad145

[CR44] Das S, Jean J-S, Chou M-L, Rathod J, Liu C-C (2016) Arsenite-oxidizing bacteria exhibiting plant growth promoting traits isolated from the rhizosphere of *Oryza sativa* L.: implications for mitigation of arsenic contamination in paddies. J Hazard Mater 302:10–18. 10.1016/j.jhazmat.2015.09.044 26448489 10.1016/j.jhazmat.2015.09.044

[CR45] Dash HR, Sahu M, Mallick B, Das S (2017) Functional efficiency of MerA protein among diverse mercury resistant bacteria for efficient use in bioremediation of inorganic mercury. Biochimie 142:207–215. 10.1016/j.biochi.2017.09.016 28966143 10.1016/j.biochi.2017.09.016

[CR46] De J, Ramaiah N, Vardanyan L (2008) Detoxification of toxic heavy metals by marine bacteria highly resistant to mercury. Mar Biotechnol 10:471–477. 10.1007/s10126-008-9083-z 10.1007/s10126-008-9083-z 18288535

[CR47] DeFlorio W, Liu S, White AR, Taylor TM, Cisneros-Zevallos L, Min Y, Scholar EM (2021) Recent developments in antimicrobial and antifouling coatings to reduce or prevent contamination and cross-contamination of food contact surfaces by bacteria. Compr Rev Food Sci Food Saf 20(3):3093–3134. 10.1111/1541-4337.12750 33949079 10.1111/1541-4337.12750

[CR206] Dhar K, Venkateswarlu K, Megharaj M (2023) Anoxygenic phototrophic purple non-sulfur bacteria: tool for bioremediation of hazardous environmental pollutants. World J Microbiol Biotechnol 39:283. 10.1007/s11274-023-03729-7 10.1007/s11274-023-03729-7 PMC10439078 37594588

[CR48] Dieppois G, Opota O, Lalucat J, Lemaitre B (2015) *Pseudomonas entomophila*: a versatile bacterium with entomopathogenic properties. In: Ramos J-L, Goldberg JB, Filloux A (eds) Pseudomonas. Springer, Netherlands, pp 25–49

[CR49] Dobrzyński J, Kulkova I, Jakubowska Z, Naziębło A, Wróbel B (2024a) *Pseudomonas* sp. G31 and *Azotobacter* sp. PBC2 changed structure of bacterial community and modestly promoted growth of oilseed rape. Int J Mol Sci 25:13168. 10.3390/ijms252313168 39684878 10.3390/ijms252313168 PMC11642319

[CR50] Dobrzyński J, Kulkova I, Jakubowska Z, Wróbel B (2024b) Non-native PGPB consortium altered the rhizobacterial community and slightly stimulated the growth of winter oilseed rape (*Brassica napus* L.) under field conditions. Microb Ecol 87:168. 10.1007/s00248-024-02471-3 10.1007/s00248-024-02471-3 PMC11711131 39774713

[CR51] Dobrzyński J, Kulkova I, Jakubowska Z, Wróbel B (2025) Non-native PGPB consortium consisting of *Pseudomonas* sp. G31 and *Azotobacter* sp. PBC2 promoted winter wheat growth and slightly altered the native bacterial community. Sci Rep 15:3248. 10.1038/s41598-025-86820-3 39863679 10.1038/s41598-025-86820-3 PMC11762297

[CR52] Doughari HJ, Ndakidemi PA, Human IS, Benade S (2011) The ecology, biology and pathogenesis of *Acinetobacter* spp.: an overview. Microb Environ 26:101–112. 10.1264/jsme2.ME10179 10.1264/jsme2.me10179 21502736

[CR53] Dutta A, Mukherjee SK, Hossain ST (2024) Characterization of As(III)-oxidizing bacteria *Acinetobacter* sp. TMKU7 having plant growth promoting features for possible application in arsenic-contaminated crop field. Bioremed J 28:457–471. 10.1080/10889868.2023.2298326

[CR203] Emerson D, Fleming EJ, McBeth JM (2010) Iron-oxidizing bacteria: An environmental and genomic perspective. Annu Rev Microbiol 64:561–583. 10.1146/annurev.micro.112408.134208 10.1146/annurev.micro.112408.134208 20565252

[CR54] Essahale A, Malki M, Marín I, Moumni M (2012) Hexavalent chromium reduction and accumulation by *Acinetobacter* AB1 isolated irom Fez tanneries in Morocco. Indian J Microbiol 52:48–53. 10.1007/s12088-011-0187-1 23459068 10.1007/s12088-011-0187-1 PMC3298598

[CR55] Etesami H (2018) Bacterial mediated alleviation of heavy metal stress and decreased accumulation of metals in plant tissues: mechanisms and future prospects. Ecotoxicol Environ Saf 147:175–191. 10.1016/j.ecoenv.2017.08.032 28843189 10.1016/j.ecoenv.2017.08.032

[CR56] Fakhar A, Gul B, Gurmani AR, Khan SM, Ali S, Sultan T, Chaudhary HJ, Rafique M, Rizwan M (2022) Heavy metal remediation and resistance mechanism of *Aeromonas*, *Bacillus*, and *Pseudomonas*: a review. Crit Rev Environ Sci Technol 52:1868–1914. 10.1080/10643389.2020.1863112

[CR57] Fan H, Su C, Wang Y, Yai J, Zhao K, Wang Y, Wang G (2008) Sedimentary arsenite-oxidizing and arsenate-reducing bacteria associated with high arsenic groundwater from Shanyin, Northwestern China. J Appl Microbiol 105:529–539. 10.1111/j.1365-2672.2008.03790.x 18397256 10.1111/j.1365-2672.2008.03790.x

[CR58] Franco MW, Mendes LA, Windmöller CC, Moura KAF, Oliveira LAG, Barbosa FAR (2018) Mercury methylation capacity and removal of Hg species from aqueous medium by cyanobacteria. Water Air Soil Pollut 229:127. 10.1007/s11270-018-3782-5

[CR210] François F, Lombard C, Guigner J-M, Soreau P, Brian-Jaisson F, Martino G, Vandervennet M, Garcia D, Molinier A-L, Pignol D, Peduzzi J, Zirah S, Rebuffat S (2012) Isolation and characterization of environmental bacteria capable of extracellular biosorption of mercury. Appl Environ Microbiol 78:1097–1106. 10.1128/AEM.06522-11 10.1128/AEM.06522-11 PMC3273009 22156431

[CR59] Gamalero E, Lingua G, Berta G, Glick BR (2009) Beneficial role of plant growth promoting bacteria and arbuscular mycorrhizal fungi on plant responses to heavy metal stress. Can J Microbiol 55:501–514. 10.1139/W09-010 19483778 10.1139/w09-010

[CR60] Ganguli A, Tripathi AK (1999) Survival and chromate reducing ability of *Pseudomonas aeruginosa* in industrial effluents. Lett Appl Microbiol 28:76–80. 10.1046/j.1365-2672.1999.00457.x 10030037 10.1046/j.1365-2672.1999.00457.x

[CR61] Gani M, Rao S, Miller M, Scoular S (2019) *Pseudomonas mendocina* bacteremia: a case study and review of literature. Am J Case Rep 20:453–458. 10.12659/AJCR.914360 30948701 10.12659/AJCR.914360 PMC6463785

[CR62] Gao J, Wu S, Liu Y, Wu S, Jiang C, Li X, Wang R, Bai Z, Zhuang G, Zhuang X (2020) Characterization and transcriptomic analysis of a highly Cr(VI)-resistant and -reductive plant-growth-promoting rhizobacterium *Stenotrophomonas rhizophila* DSM14405T. Environ Pollut 263:114622. 10.1016/j.envpol.2020.114622

[CR63] Garg SK, Tripathi M, Singh SK, Singh A (2013) Pentachlorophenol dechlorination and simultaneous Cr^6+^ reduction by *Pseudomonas putida* SKG-1 MTCC (10510): characterization of PCP dechlorination products, bacterial structure, and functional groups. Environ Sci Pollut Res 20:2288–2304. 10.1007/s11356-012-1101-z 10.1007/s11356-012-1101-z 22864755

[CR64] Ge S, Ge SC (2016) Simultaneous Cr(VI) reduction and Zn(II) biosorption by *Stenotrophomonas* sp. and constitutive expression of related genes. Biotechnol Lett 38:877–884. 10.1007/s10529-016-2057-8 26861853 10.1007/s10529-016-2057-8

[CR65] Ghosh P, Rathinasabapathi B, Teplitski M, Ma LQ (2015) Bacterial ability in AsIII oxidation and AsV reduction: relation to arsenic tolerance, P uptake, and siderophore production. Chemosphere 138:995–1000. 10.1016/j.chemosphere.2014.12.046 25576133 10.1016/j.chemosphere.2014.12.046

[CR66] Giovanella P, Cabral L, Bento FM, Gianello C, Oliveira Camargo FA (2016) Mercury (II) removal by resistant bacterial isolates and mercuric (II) reductase activity in a new strain of *Pseudomonas* sp. B50A. N Biotechnol 33:216–223. 10.1016/j.nbt.2015.05.006 26051077 10.1016/j.nbt.2015.05.006

[CR67] González PS, Ambrosio LF, Paisio CE, Talano MA, Medina MI, Agostini E (2014) Chromium (VI) remediation by a native strain: effect of environmental conditions and removal mechanisms involved. Environ Sci Pollut Res 21:13551–13559. 10.1007/s11356-014-3311-z 10.1007/s11356-014-3311-z 25023657

[CR68] Gounot A-M (1994) Microbial oxidation and reduction of manganese: consequences in groundwater and applications. FEMS Microbiol Rev 14:339–350 7917421 10.1111/j.1574-6976.1994.tb00108.x

[CR69] Gunasundari D, Muthukumar K (2013) Simultaneous Cr(VI) reduction and phenol degradation using *Stenotrophomonas* sp. isolated from tannery effluent contaminated soil. Environ Sci Pollut Res 20:6563–6573. 10.1007/s11356-013-1718-6 10.1007/s11356-013-1718-6 23608988

[CR70] Hamamura N, Fukushima K, Itai T (2013) Identification of antimony- and arsenic-oxidizing bacteria associated with antimony mine tailing. Microb Environ 28:257–263. 10.1264/jsme2.ME12217 10.1264/jsme2.ME12217 PMC4070671 23666539

[CR71] Handley KM, Héry M, Lloyd JR (2009) Redox cycling of arsenic by the hydrothermal marine bacterium *Marinobacter santoriniensis*. Environ Microbiol 11:1601–1611. 10.1111/j.1462-2920.2009.01890.x 19226300 10.1111/j.1462-2920.2009.01890.x

[CR72] He Z, Gao F, Sha T, Hu Y, He C (2009) Isolation and characterization of a Cr(VI)-reduction *Ochrobactrum* sp. strain CSCr-3 from chromium landfill. J Hazard Mater 163:869–873. 10.1016/j.jhazmat.2008.07.041 18722054 10.1016/j.jhazmat.2008.07.041

[CR73] He D, Zheng M, Ma T, Li C, Ni J (2015) Interaction of Cr(VI) reduction and denitrification by strain *Pseudomonas aeruginosa* PCN-2 under aerobic conditions. Biores Technol 185:346–352. 10.1016/j.biortech.2015.02.109 10.1016/j.biortech.2015.02.109 25795449

[CR74] He X, Xiao W, Zeng J, Tang J, Wang L (2023) Detoxification and removal of arsenite by *Pseudomonas* sp. SMS11: oxidation, biosorption and bioaccumulation. J Environ Manage 336:117641. 10.1016/j.jenvman.2023.117641 36868151 10.1016/j.jenvman.2023.117641

[CR75] Hernandez-Maldonado J, Sanchez-Sedillo B, Stoneburner B, Boren A, Miller L, McCann S, Rosen M, Oremland RS, Saltikov CV (2017) The genetic basis of anoxygenic photosynthetic arsenite oxidation. Environ Microbiol 19(1):130–141. 10.1111/1462-2920.13509 27555453 10.1111/1462-2920.13509 PMC5967609

[CR76] Hosseinkhani B, Emtiazi G (2011) Synthesis and characterization of a novel extracellular biogenic manganese oxide (bixbyite-like Mn_2_O_3_) nanoparticle by isolated *Acinetobacter* sp. Curr Microbiol 63:300–305. 10.1007/s00284-011-9971-8 21761221 10.1007/s00284-011-9971-8

[CR77] Hu L, Liu B, Li S, Zhong H, He Z (2021) Study on the oxidative stress and transcriptional level in Cr(VI) and Hg(II) reducing strain *Acinetobacter indicus* yy-1 isolated from chromium-contaminated soil. Chemosphere 269:128741. 10.1016/j.chemosphere.2020.128741 33127119 10.1016/j.chemosphere.2020.128741

[CR78] Ibrahim ASS, El-Tayeb MA, Elbadawi YB, Al-Salamah AA, Antranikian G (2012) Hexavalent chromate reduction by alkaliphilic *Amphibacillus* sp. KSUCr3 is mediated by copper-dependent membrane-associated Cr(VI) reductase. Extremophiles 16:659–668. 10.1007/s00792-012-0464-x 22669507 10.1007/s00792-012-0464-x

[CR79] Ito A, Miura J, Ishikawa N, Umita T (2012) Biological oxidation of arsenite in synthetic groundwater using immobilised bacteria. Water Res 46:4825–4831. 10.1016/j.watres.2012.06.013 22760058 10.1016/j.watres.2012.06.013

[CR80] Jan AT, Murtaza I, Ali A, Haq QMohd R (2009) Mercury pollution: an emerging problem and potential bacterial remediation strategies. World J Microbiol Biotechnol 25:1529–1537. 10.1007/s11274-009-0050-2

[CR81] Jung J, Park W (2015) *Acinetobacter* species as model microorganisms in environmental microbiology: current state and perspectives. Appl Microbiol Biotechnol 99:2533–2548. 10.1007/s00253-015-6439-y 25693672 10.1007/s00253-015-6439-y

[CR82] Kang S-Y, Lee J-U, Kim K-W (2007) Biosorption of Cr(III) and Cr(VI) onto the cell surface of *Pseudomonas aeruginosa*. Biochem Eng J 36:54–58. 10.1016/j.bej.2006.06.005

[CR83] Karn SK, Pan X (2017) Bacterial oxidation and stabilization of As(III) in soil. Environ Eng Sci 34:158–164. 10.1089/ees.2015.0390

[CR84] Katsoyiannis IA, Zouboulis AI (2004) Application of biological processes for the removal of arsenic from groundwaters. Water Res 38:17–26. 10.1016/j.watres.2003.09.011 14630099 10.1016/j.watres.2003.09.011

[CR85] Katsoyiannis IA, Zouboulis AI (2006) Use of iron- and manganese-oxidizing bacteria for the combined removal of iron, manganese and arsenic from contaminated groundwater. Water Qual Res J 41:117–129. 10.2166/wqrj.2006.014

[CR86] Kavita B, Keharia H (2012) Reduction of hexavalent chromium by *Ochrobactrum intermedium* BCR400 isolated from a chromium-contaminated soil. 3 Biotech 2:79–87. 10.1007/s13205-011-0038-0 22582159 10.1007/s13205-011-0038-0 PMC3339614

[CR87] Kitjanukit S, Takamatsu K, Okibe N (2019) Natural attenuation of Mn(II) in metal refinery wastewater: microbial community structure analysis and isolation of a new Mn(II)-oxidizing bacterium *Pseudomonas* sp. SK3. Water 11:507. 10.3390/w11030507

[CR88] Komal T, Mustafa M, Ali Z, Kazi AG (2015) Heavy metal uptake and transport in plants. In: Sherameti I, Varma A (eds) Heavy metal contamination of soils, soil biology. Springer International Publishing, Cham, pp 181–194

[CR89] Kristanti RA, Hadibarata T, Syafrudin M, Yılmaz M, Abdullah S (2022) Microbiological contaminants in drinking water: current status and challenges. Water Air Soil Pollut 233(8):299. 10.1007/s11270-022-05698-3

[CR202] Lakshmanan V, Shantharaj D, Li G, Seyffert AL, Sherrier DJ, Bais HP (2015) A natural rice rhizospheric bacterium abates arsenic accumulation in rice (*Oryza sativa* L). Planta 242:1037–1050. 10.1007/s00425-015-2340-2 10.1007/s00425-015-2340-2 26059607

[CR90] Li W, Yang Y, Achal V (2022) Biochemical composite material using corncob powder as a carrier material for ureolytic bacteria in soil cadmium immobilization. Sci Total Environ 802:149802. 10.1016/j.scitotenv.2021.149802 34464799 10.1016/j.scitotenv.2021.149802

[CR211] Lalucat J, Gomila M, Mulet M, Zaruma A, García-Valdés E (2022) Past, present and future of the boundaries of the *Pseudomonas* genus: Proposal of *Stutzerimonas* gen. nov. Syst Appl Microbiol 45:126289. 10.1016/j.syapm.2021.126289 10.1016/j.syapm.2021.126289 34920232

[CR91] Li X, Li J, Zhao Q, Qiao L, Wang L, Yu C (2023) Physiological, biochemical, and genomic elucidation of the *Ensifer adhaerens* M8 strain with simultaneous arsenic oxidation and chromium reduction. J Hazard Mater 441:129862. 10.1016/j.jhazmat.2022.129862 36084460 10.1016/j.jhazmat.2022.129862

[CR92] Li K, Yu K, Huang Z, Liu X, Mei L, Ren X, Bai X, Gao H, Sun Z, Liu X, Wang D (2024) *Stenotrophomonas maltophilia* complex: insights into evolutionary relationships, global distribution and pathogenicity. Front Cell Infect Microbiol 13:1325379. 10.3389/fcimb.2023.1325379 38268792 10.3389/fcimb.2023.1325379 PMC10806987

[CR93] Liao S, Zhou J, Wang H, Chen X, Wang H, Wang G (2013) Arsenite oxidation using biogenic manganese oxides produced by a deep-sea manganese-oxidizing bacterium, *Marinobacter* sp. MnI7-9. Geomicrobiol J 30:150–159. 10.1080/01490451.2011.654379

[CR94] Liu M, Wang S, Yang M, Ning X, Nan Z (2022) Experimental study on treatment of heavy metal–contaminated soil by manganese-oxidizing bacteria. Environ Sci Pollut Res 29:5526–5540. 10.1007/s11356-021-15475-0 10.1007/s11356-021-15475-0 34424469

[CR95] Lu X, Zhang Y, Liu C, Wu M, Wang H (2018) Characterization of the antimonite- and arsenite-oxidizing bacterium *Bosea* sp. AS-1 and its potential application in arsenic removal. J Hazard Mat 359:527–534. 10.1016/j.jhazmat.2018.07.112 10.1016/j.jhazmat.2018.07.112 30086523

[CR96] Macur RE, Jackson CR, Botero LM, Mcdermott TR, Inskeep WP (2004) Bacterial populations associated with the oxidation and reduction of arsenic in an unsaturated soil. Environ Sci Technol 38:104–111. 10.1021/es034455a 14740724 10.1021/es034455a

[CR97] Mahbub KR, Krishnan K, Megharaj M, Naidu R (2016a) Bioremediation potential of a highly mercury resistant bacterial strain *Sphingobium* SA2 isolated from contaminated soil. Chemosphere 144:330–337. 10.1016/j.chemosphere.2015.08.061 26378869 10.1016/j.chemosphere.2015.08.061

[CR98] Mahbub KR, Krishnan K, Naidu R, Megharaj M (2016b) Mercury resistance and volatilization by *Pseudoxanthomonas* sp. SE1 isolated from soil. Environ Technol Innov 6:94–104. 10.1016/j.eti.2016.08.001

[CR99] Malaviya P, Singh A (2016) Bioremediation of chromium solutions and chromium containing wastewaters. Crit Rev Microbiol 42:607–633. 10.3109/1040841X.2014.974501 25358056 10.3109/1040841X.2014.974501

[CR100] Manfredini A, Malusà E, Costa C, Pallottino F, Mocali S, Pinzari F, Canfora L (2021) Current methods, common practices, and perspectives in tracking and monitoring bioinoculants in soil. Front Microbiol 12:698491. 10.3389/fmicb.2021.698491 34531836 10.3389/fmicb.2021.698491 PMC8438429

[CR101] Mao Q, Wei D, Yan B, Luo S, Seviour TW, Wei Z, Xie X, Luo L (2022) Removal of manganese in acidic solutions utilizing *Achromobacter* sp. strain QBM-4 isolated from mine drainage. Process Saf Environ Prot 165:920–928. 10.1016/j.psep.2022.04.002

[CR102] Mello IS, Targanski S, Pietro-Souza W, Frutuoso Stachack FF, Terezo AJ, Soares MA (2020) Endophytic bacteria stimulate mercury phytoremediation by modulating its bioaccumulation and volatilization. Ecotoxicol Environ Saf 202:110818. 10.1016/j.ecoenv.2020.110818 32590206 10.1016/j.ecoenv.2020.110818

[CR103] Miao L, Chen S, Yang H, Hong Y, Sun L, Yang J, Cheng Y (2023) Enhanced bioremediation of triclocarban-contaminated soil by Rhodococcus rhodochrous BX2 and Pseudomonas sp. LY-1 immobilized on biochar and microbial community response. Front Microbiol 14:1168902. 10.3389/fmicb.2023.1168902 37065135 10.3389/fmicb.2023.1168902 PMC10098447

[CR104] Millaleo R, Reyes- Diaz M, Ivanov AG, Mora ML, Alberdi M (2010) Manganese as essential and toxic element for plants: transport, accumulation and resistance mechanisms. J Soil Sci Plant Nutr 10:470–481. 10.4067/S0718-95162010000200008

[CR105] Min X, Zhang K, Chen J, Chai L, Lin Z, Zou L, Liu W, Ding C, Shi Y (2024) Bacteria-driven copper redox reaction coupled electron transfer from Cr(VI) to Cr(III): a new and alternate mechanism of Cr(VI) bioreduction. J Hazard Mater 461:132485. 10.1016/j.jhazmat.2023.132485 37714006 10.1016/j.jhazmat.2023.132485

[CR106] Miranda-Carrazco A, Vigueras-Cortés JM, Villa-Tanaca L, Hernández-Rodríguez C (2018) Cyanotrophic and arsenic oxidizing activities of *Pseudomonas mendocina* P6115 isolated from mine tailings containing high cyanide concentration. Arch Microbiol 200:1037–1048. 10.1007/s00203-018-1514-2 29644379 10.1007/s00203-018-1514-2

[CR107] Misra P, Archana US, Srivastava AK (2022) *Pseudomonas* for sustainable agricultural ecosystem. Microbial syntrophy-mediated eco-enterprising. Elsevier, pp 209–223

[CR108] Mondaca MA, Campos V, Moraga R, Zaror CA (2002) Chromate reduction in *Serratia marcescens* isolated from tnnery effluent and potential application for bioremediation of chromate pollution. Sci World J 2:972–977. 10.1100/tsw.2002.154 10.1100/tsw.2002.154 PMC6009734 12805951

[CR109] Mukkata K, Kantachote D, Wittayaweerasak B, Techkarnjanaruk S, Mallavarapu M, Naidu R (2015) Distribution of mercury in shrimp ponds and volatilization of Hg by isolated resistant purple nonsulfur bacteria. Water Air Soil Pollut 226:148. 10.1007/s11270-015-2418-2

[CR110] Murray KJ, Mozafarzadeh ML, Tebo BM (2005) Cr(III) oxidation and Cr toxicity in cultures of the manganese(II)-oxidizing *Pseudomonas putida* strain GB-1. Geomicrobiol J 22:151–159. 10.1080/01490450590945988

[CR111] Narayani M, Vidya Shetty K (2012) Characteristics of a novel *Acinetobacter* sp. and its kinetics in hexavalent chromium bioreduction. J Microbiol Biotechnol 22:690–698. 10.4014/jmb.1110.10073 22561865 10.4014/jmb.1110.10073

[CR112] Nguyen VK, Ha M-G, Kang HY, Nguyen DD (2020) Biological manganese removal by novel halotolerant bacteria isolated from river water. Biomolecules 10:941. 10.3390/biom10060941 32580482 10.3390/biom10060941 PMC7356865

[CR201] Nookongbut P, Kantachote D, Megharaj M, Naidu R (2018) Reduction in arsenic toxicity and uptake in rice (*Oryza sativa* L.) by Asresistant purple nonsulfur bacteria. Environ Sci Pollut Res 25:36530–36544. 10.1007/s11356-018-3568-8 10.1007/s11356-018-3568-8 30374717

[CR205] Norton JM (2014) Diversity and environmental distribution of ammonia-oxidizing bacteria. In: Ward BB, Arp DJ, Klotz MG (eds) Nitrification. ASM Press, Washington, DC, USA, pp 39–55

[CR113] Opperman DJ, Van Heerden E (2007) Aerobic Cr(VI) reduction by *Thermus scotoductus* strain SA-01: Cr(VI) reduction by *Thermus scotoductus*. J Appl Microbiol 103:1907–1913. 10.1111/j.1365-2672.2007.03429.x 17953600 10.1111/j.1365-2672.2007.03429.x

[CR114] Osborn AM, Bruce KD, Strike P, Ritchie DA (1997) Distribution, diversity and evolution of the bacterial mercury resistance (*mer*) operon. FEMS Microbiol Rev 19:239–262. 10.1111/j.1574-6976.1997.tb00300.x 9167257 10.1111/j.1574-6976.1997.tb00300.x

[CR115] Ospino MC, Kojima H, Fukui M (2019) Arsenite oxidation by a newly isolated Betaproteobacterium possessing arx genes and diversity of the arx gene cluster in bacterial genomes. Front Microbiol 10:1210. 10.3389/fmicb.2019.01210 31191509 10.3389/fmicb.2019.01210 PMC6549141

[CR116] Ozturk S, Kaya T, Aslim B, Tan S (2012) Removal and reduction of chromium by *Pseudomonas* spp. and their correlation to rhamnolipid production. J Hazard Mater. 10.1016/j.jhazmat.2012.06.038 22790393 10.1016/j.jhazmat.2012.06.038

[CR117] Pal A, Dutta S, Paul AK (2005) Reduction of hexavalent chromium by cell-free extract of *Bacillus sphaericus* AND 303 isolated from serpentine soil. Curr Microbiol 51:327–330. 10.1007/s00284-005-0048-4 16163455 10.1007/s00284-005-0048-4

[CR118] Perez-Vazquez A, Barcielam P, Prieto MA (2024) In situ and ex situ bioremediation of different persistent soil pollutants as agroecology tool. Processes 12(10):2223. 10.3390/pr12102223

[CR119] Petrus AK, Rutner C, Liu S, Wang Y, Wiatrowski HA (2015) Mercury reduction and methyl mercury degradation by the soil bacterium *Xanthobacter autotrophicus* Py2. Appl Environ Microbiol 81(22):7833–7838. 10.1128/AEM.01982-15 26341208 10.1128/AEM.01982-15 PMC4616949

[CR120] Piazza A, Ciancio Casalini L, Pacini VA, Sanguinetti G, Ottado J, Gottig N (2019) Environmental bacteria involved in manganese(II) oxidation and removal from groundwater. Front Microbiol 10:119. 10.3389/fmicb.2019.00119 30853942 10.3389/fmicb.2019.00119 PMC6396730

[CR121] Płociniczak T, Pacwa-Płociniczak M, Kwaśniewski M, Chwiałkowska K, Piotrowska-Seget Z (2020) Response of rhizospheric and endophytic bacterial communities of white mustard (Sinapis alba) to bioaugmentation of soil with the Pseudomonas sp. H15 strain. Ecotoxicol Environ Saf 194:110434. 10.1016/j.ecoenv.2020.110434 32155483 10.1016/j.ecoenv.2020.110434

[CR122] Pradhan SK, Singh NR, Rath BP, Thatoi H (2016) Bacterial chromate reduction: a review of important genomic, proteomic, and bioinformatic analysis. Crit Rev Environ Sci Technol 46:1659–1703. 10.1080/10643389.2016.1258912

[CR123] Pulimi M (2012) Enhancing the hexavalent chromium bioremediation potential of *Acinetobacter junii* VITSUKMW2 using statistical design experiments. J Microbiol Biotechnol 22:1767–1775. 10.4014/jmb.1203.03063 23221541 10.4014/jmb.1203.03063

[CR124] Puschenreiter M, Horak O, Friesl W, Hartl W (2005) Low-cost agricultural measures to reduce heavy metal transfer into the food chain - a review. Plant Soil Environ 51(1):1–11. 10.17221/3549-PSE

[CR125] Queiroz PS, Barboza NR, Cordeiro MM, Leão VA, Guerra-Sá R (2018) Rich growth medium promotes an increased on Mn(II) removal and manganese oxide production by *Serratia marcescens* strains isolates from wastewater. Biochem Eng J 140:148–156. 10.1016/j.bej.2018.09.018

[CR126] Ram Talib NS, Halmi MIE, Abd Ghani SS, Zaidan UH, Shukor MYA (2019) Artificial neural networks (ANNs) and response surface methodology (RSM) approach for modelling the optimization of chromium (VI) reduction by newly isolated *Acinetobacter radioresistens* strain NS-MIE from agricultural soil. BioMed Res Int 2019:1–14. 10.1155/2019/5785387 10.1155/2019/5785387 PMC6556361 31240217

[CR204] Ranadev P, Revanna A, Bagyaraj DJ, Shinde AH (2023) Sulfur oxidizing bacteria in agro ecosystem and its role in plant productivity a review. J Appl Microbiol 134:lxad161. 10.1093/jambio/lxad161 10.1093/jambio/lxad161 37491695

[CR127] Reynolds D, Kollef M (2021) The epidemiology and pathogenesis and treatment of *Pseudomonas aeruginosa* infections: an update. Drugs 81:2117–2131. 10.1007/s40265-021-01635-6 34743315 10.1007/s40265-021-01635-6 PMC8572145

[CR128] Ryan RP, Monchy S, Cardinale M, Taghavi S, Crossman L, Avison MB, Berg G, Van Der Lelie D, Dow JM (2009) The versatility and adaptation of bacteria from the genus *Stenotrophomonas*. Nat Rev Microbiol 7:514–525. 10.1038/nrmicro2163 19528958 10.1038/nrmicro2163

[CR129] Sagar S, Dwivedi A, Yadav S, Tripathi M, Kaistha SD (2012) Hexavalent chromium reduction and plant growth promotion by *Staphylococcus arlettae* strain Cr11. Chemosphere 86:847–852. 10.1016/j.chemosphere.2011.11.031 22169713 10.1016/j.chemosphere.2011.11.031

[CR130] Salmassi TM, Venkateswaren K, Satomi M, Newman DK, Hering JG (2002) Oxidation of arsenite by *Agrobacterium albertimagni*, AOL15, sp. nov., isolated from hot creek. California. Geomicrobiol J 19:53–66. 10.1080/014904502317246165

[CR131] Samantaray DP, Mishra BB (2012) Effect of metal on hexavalent chromium reduction by *Acinetobacter calcoaceticus*. Bioscan 7:627–629

[CR132] Santamaría J, Toranzos GA (2003) Enteric pathogens and soil: a short review. Int Microbiol 6:5–9. 10.1007/s10123-003-0096-111 12730707 10.1007/s10123-003-0096-1

[CR133] Sarangi A, Krishnan C (2008) Comparison of in vitro Cr(VI) reduction by CFEs of chromate resistant bacteria isolated from chromate contaminated soil. Bioresour Technol 99:4130–4137. 10.1016/j.biortech.2007.08.059 17920879 10.1016/j.biortech.2007.08.059

[CR134] Sathishkumar K, Murugan K, Benelli G, Higuchi A, Rajasekar A (2017) Bioreduction of hexavalent chromium by *Pseudomonas stutzeri* L1 and *Acinetobacter baumannii* L2. Ann Microbiol 67:91–98. 10.1007/s13213-016-1240-4

[CR135] Sayel H, Bahafid W, Tahri Joutey N, Derraz K, Fikri Benbrahim K, Ibnsouda Koraichi S, El Ghachtouli N (2012) Cr(VI) reduction by *Enterococcus gallinarum* isolated from tannery waste-contaminated soil. Ann Microbiol 62:1269–1277. 10.1007/s13213-011-0372-9

[CR136] Sevak P, Pushkar B, Mazumdar S (2023) Mechanistic evaluation of chromium bioremediation in *Acinetobacter junii* strain b2w: a proteomic approach. J Environ Manage 328:116978. 10.1016/j.jenvman.2022.116978 36521220 10.1016/j.jenvman.2022.116978

[CR137] Sher S, Rehman A (2019) Use of heavy metals resistant bacteria—a strategy for arsenic bioremediation. Appl Microbiol Biotechnol 103:6007–6021. 10.1007/s00253-019-09933-6 31209527 10.1007/s00253-019-09933-6

[CR138] Shi Y, Chai L, Yang Z, Jing Q, Chen R, Chen Y (2012) Identification and hexavalent chromium reduction characteristics of *Pannonibacter phragmitetus*. Bioprocess Biosyst Eng 35:843–850. 10.1007/s00449-011-0668-y 22179413 10.1007/s00449-011-0668-y

[CR139] Shi Y, Wang Z, Li H, Yan Z, Meng Z, Liu C, Chen J, Duan C (2023) Resistance mechanisms and remediation potential of hexavalent chromium in *Pseudomonas* sp. strain AN-B15. Ecotoxicol Environ Saf 250:114498. 10.1016/j.ecoenv.2023.114498 36608568 10.1016/j.ecoenv.2023.114498

[CR140] Singh JS, Abhilash PC, Singh HB, Singh RP, Singh DP (2011) Genetically engineered bacteria: an emerging tool for environmental remediation and future research perspectives. Gene 480(1–2):1–9. 10.1016/j.gene.2011.03.001 21402131 10.1016/j.gene.2011.03.001

[CR141] Singh AL, Singh VK, Yadav A (2016) Arsenic sequestration by manganese-oxidizing *Acinetobacter* sp. Indian J Biotechnol 15:525–530

[CR142] Singh P, Montano A, Bostick A (2021) Rapid severe sepsis from *Pseudomonas fluorescens/putida* bacteremia due to skin and soft tissue infection—a case report. Ann Med Surg 70:102845. 10.1016/j.amsu.2021.102845 10.1016/j.amsu.2021.102845 PMC8437775 34540222

[CR143] Smith T, Pitts K, McGarvey JA, Summers AO (1998) Bacterial oxidation of mercury metal vapor, Hg(0). Appl Environ Microbiol 64:1328–1332 9546169 10.1128/aem.64.4.1328-1332.1998 PMC106150

[CR144] Sobecky PA, Coombs JM (2009) Horizontal gene transfer in metal and radionuclide contaminated soils. In: Gogarten MB, Gogarten JP, Olendzenski LC (eds) Horizontal gene transfer: Methods in Molecular Biology, vol 532. Humana Press 10.1007/978-1-60327-853-9_26 19271201

[CR145] Some S, Mondal R, Mitra D, Jain D, Verma D, Das S (2021) Microbial pollution of water with special reference to coliform bacteria and their nexus with environment. Energy Nexus 1:100008. 10.1016/j.nexus.2021.100008

[CR146] Song F, Zhang G, Xu X, Polyak SW, Zhang K, Li H, Yang N (2022) Role of intracellular energy metabolism in Mn(II) removal by the novel bacterium *Stenotrophomonas* sp. MNB17. Chemosphere 308(2):136435. 10.1016/j.chemosphere.2022.136435 36113658 10.1016/j.chemosphere.2022.136435

[CR147] Su JF, Zheng SC, Huang TL, Ma F, Shao SC, Yang SF, Zhang LN (2016) Simultaneous removal of Mn(II) and nitrate by the manganese-oxidizing bacterium *Acinetobacter* sp. SZ28 in anaerobic conditions. Geomicrobiol J 33:586–591. 10.1080/01490451.2015.1063024

[CR148] Sugio T, Fujii M, Takeuchi F, Negishi A, Maeda T, Kamimura K (2003) Volatilization of mercury by an iron oxidation enzyme system in a highly mercury-resistant *Acidithiobacillus ferrooxidans* strain MON-1. Biosci Biotechnol Biochem 67(7):1537–1544. 10.1271/bbb.67.1537 12913298 10.1271/bbb.67.1537

[CR149] Svensson-Stadler LA, Mihaylova SA, Moore ERB (2012) *Stenotrophomonas* interspecies differentiation and identification by *gyrB* sequence analysis. FEMS Microbiol Lett 327:15–24. 10.1111/j.1574-6968.2011.02452.x 22092789 10.1111/j.1574-6968.2011.02452.x

[CR150] Szyttenholm J, Chaspoul F, Bauzan M, Ducluzeau A-L, Chehade MH, Pierrel F et al (2020) The controversy on the ancestral arsenite oxidizing enzyme; deducing evolutionary histories with phylogeny and thermodynamics. BBA—Bioenergetics 1861:148252. 10.1016/j.bbabio.2020.148252 32569664 10.1016/j.bbabio.2020.148252

[CR151] Tan H, Wang C, Zeng G, Luo Y, Li H, Xu H (2020) Bioreduction and biosorption of Cr(VI) by a novel *Bacillus* sp. CRB-B1 strain. J Hazard Mater 386:121628. 10.1016/j.jhazmat.2019.121628 31744729 10.1016/j.jhazmat.2019.121628

[CR152] Tang W, Xia J, Zeng X, Wu L, Ye G (2014) Biological characteristics and oxidation mechanism of a new manganese-oxidizing bacteria FM-2. Niomed Mater Eng 24:703–709. 10.3233/BME-130858 10.3233/BME-130858 24211955

[CR153] Tang W, Gong J, Wu L, Li Y, Zhang M, Zeng X (2016) DGGE diversity of manganese mine samples and isolation of a *Lysinibacillus* sp. efficient in removal of high Mn (II) concentrations. Chemosphere 165:277–283. 10.1016/j.chemosphere.2016.08.134 27657820 10.1016/j.chemosphere.2016.08.134

[CR154] Tchounwou PB, Yedjou CG, Patlolla AK, Sutton DJ (2012) Heavy metal toxicity and the environment. In: Luch A (ed) Molecular, clinical and environmental toxicology: experientia supplementum. Springer, Basel, pp 133–164 10.1007/978-3-7643-8340-4_6 PMC4144270 22945569

[CR155] Thacker U, Parikh R, Shouche Y, Madamwar D (2007) Reduction of chromate by cell-free extract of *Brucella* sp. isolated from Cr(VI) contaminated sites. Biores Technol 98:1541–1547. 10.1016/j.biortech.2006.06.011 10.1016/j.biortech.2006.06.011 16931000

[CR156] Thatoi H, Das S, Mishra J, Rath BP, Das N (2014) Bacterial chromate reductase, a potential enzyme for bioremediation of hexavalent chromium: a review. J Environ Manage 146:383–399. 10.1016/j.jenvman.2014.07.014 25199606 10.1016/j.jenvman.2014.07.014

[CR157] Toyoda K, Tebo BM (2013) The effect of Ca^2+^ ions and ionic strength on Mn(II) oxidation by spores of the marine *Bacillus* sp. SG-1. Geochim Cosmochim Acta 101:1–11. 10.1016/j.gca.2012.10.008 29176910 10.1016/j.gca.2012.10.008 PMC5701786

[CR158] Tripti K, Narain D, Singh DS, Shardendu S (2017) Evaluation of arsenic removal potential of arsenic resistant bacteria with the role of physiological and genomic factors. Ind J Exp Biol 55:251–261. 10.13140/RG.2.2.17430.09282

[CR159] Upadhyay S, Tarafdar A, Sinha A (2020) Assessment of *Serratia* sp. isolated from iron ore mine in hexavalent chromium reduction: kinetics, fate and variation in cellular morphology. Environ Technol 41:1117–1126. 10.1080/09593330.2018.1521875 30198414 10.1080/09593330.2018.1521875

[CR160] Valls M, de Lorenzo V (2002) Exploiting the genetic and biochemical capacities of bacteria for the remediation of heavy metal pollution. FEMS Microbiol Rev 26:327–338 12413663 10.1111/j.1574-6976.2002.tb00618.x

[CR161] Vickram AS, Shofia SI, Palanivelu J, Karishma S, Yaashikaa PR (2024) A comprehensive analysis and exploration of the recent developments in the utilization of genetically modified microorganisms for the remediation of hazardous dye pollutants. Groundw Sustain Dev. 10.1016/j.gsd.2024.101315

[CR162] Villegas-Torres MF, Bedoya-Reina OC, Salazar C, Vives-Florez MJ, Dussan J (2011) Horizontal *arsC* gene transfer among microorganisms isolated from arsenic polluted soil. Int Biodeterior Biodegrad 65(1):147–152. 10.1016/j.ibiod.2010.10.007

[CR163] Von Canstein H, Kelly S, Li Y, Wagner-Döbler I (2002) Species diversity improves the efficiency of mercury-reducing biofilms under changing environmental conditions. Appl Environ Microbiol 68:2829–2837. 10.1128/AEM.68.6.2829-2837.2002 12039739 10.1128/AEM.68.6.2829-2837.2002 PMC123942

[CR164] Wang W, Shao Z, Liu Y, Wang G (2009) Removal of multi-heavy metals using biogenic manganese oxides generated by a deep-sea sedimentary bacterium—*Brachybacterium* sp. strain Mn32. Microbiology 155:1989–1996. 10.1099/mic.0.024141-0 19383675 10.1099/mic.0.024141-0

[CR165] Wang X, Yu M, Wang L, Lin H, Li B, Xue C-X, Sun H, Zhang X-H (2020) Comparative genomic and metabolic analysis of manganese-oxidizing mechanisms in *Celeribacter manganoxidans* DY25T: its adaptation to the environment of polymetallic nodules. Genomics 112:2080–2091. 10.1016/j.ygeno.2019.12.002 31809796 10.1016/j.ygeno.2019.12.002

[CR166] Wang X, Xie G-J, Tian N, Dang C-C, Cai C, Ding J, Liu B-F, Xing D-F, Ren N-Q, Wang Q (2022) Anaerobic microbial manganese oxidation and reduction: a critical review. Sci Total Environ 822:153513. 10.1016/j.scitotenv.2022.153513 35101498 10.1016/j.scitotenv.2022.153513

[CR213] Wang H, Wu Q, Wang H, Liu F, Wu D, Wang X, Yuan Q (2025) The hidden diversity and functional potential of *Chloroflexota* genomes in arsenic and antimony co-contaminated soils. Soil Ecol Lett 7(1):240266. 10.1007/s42832-024-0266-y

[CR167] Wani PA, Wahid S, Khan MSA, Rafi N, Wahid N (2019) Investigation of the role of chromium reductase for Cr (VI) reduction by *Pseudomonas* species isolated from Cr (VI) contaminated effluent. Biotechnol Res Innov 3:38–46. 10.1016/j.biori.2019.04.001

[CR168] Weeger W, Lièvremont D, Perret M, Lagarde F, Hubert J-C, Leroy M, Lett M-C (1999) Oxidation of arsenite to arsenate by a bacterium isolated from an aquatic environment. Biometals 12:141–149. 10.1023/A:1009255012328 10406083 10.1023/a:1009255012328

[CR169] Wiatrowski HA, Ward PM, Barkay T (2006) Novel reduction of mercury(II) by mercury-sensitive dissimilatory metal reducing bacteria. Environ Sci Technol 40:6690–6696. 10.1021/es061046g 17144297 10.1021/es061046g

[CR170] Wright MH, Geszvain K, Oldham VE, Luther GW, Tebo BM (2018) Oxidative formation and removal of complexed Mn(III) by *Pseudomonas* species. Front Microbiol 9:560. 10.3389/fmicb.2018.00560 29706936 10.3389/fmicb.2018.00560 PMC5906577

[CR171] Wróbel M, Śliwakowski W, Kowalczyk P, Kramkowski K, Dobrzyński J (2023) Bioremediation of heavy metals by the genus *Bacillus*. Int J Environ Res Public Health 20:4964. 10.3390/ijerph20064964 36981874 10.3390/ijerph20064964 PMC10049623

[CR172] Wu Y, Deng B, Xu H, Kornishi H (2005) Chromium(III) oxidation coupled with microbially mediated Mn(II) oxidation. Geomicrobiol J 22:161–170. 10.1080/01490450590945997

[CR173] Wu Y-F, Chai C-W, Li Y-N, Chen J, Yuan Y, Hu G, Rosen BP, Zhang J (2021) Anaerobic As(III) oxidation coupled with nitrate reduction and attenuation of dissolved arsenic by *Noviherbaspirillum* Species. ACS Earth Space Chem 5:2115–2123. 10.1021/acsearthspacechem.1c00155

[CR174] Wu J, Kang F, Wang Z, Song L, Guan X, Zhou H (2022a) Manganese removal and product characteristics of a marine manganese-oxidizing bacterium *Bacillus* sp. FF-1. Int Microbiol 25:701–708. 10.1007/s10123-022-00254-9 35687202 10.1007/s10123-022-00254-9

[CR175] Wu R, Yao F, Li X, Shi C, Zang X, Shu X, Liu H, Zhang W (2022b) Manganese pollution and its remediation: a review of biological removal and promising combination strategies. Microorganisms 10:2411. 10.3390/microorganisms10122411 36557664 10.3390/microorganisms10122411 PMC9781601

[CR176] Yang F, Li J, Wang H, Xiao X, Bai R, Zhao F (2023) Visible light induces bacteria to produce superoxide for manganese oxidation. Front Environ Sci Eng 17:19. 10.1007/s11783-023-1619-y

[CR234] Yang Y, Xu Z, Yang L, Hu M-y, Jiang G-y, Chen J, Yang Y-c, Tian Y (2024) Ochrobactrum chromiisoli sp. nov., isolated from chromium-contaminated soil. Curr Microbiol 81:50. 10.1007/s00284-023-03562-z 10.1007/s00284-023-03562-z 38150064

[CR177] Yao Y, Hu L, Li S, Zeng Q, Zhong H, He Z (2020) Exploration on the bioreduction mechanisms of Cr(VI) and Hg(II) by a newly isolated bacterial strain *Pseudomonas umsongensis* CY-1. Ecotoxicol Environ Saf 201:110850. 10.1016/j.ecoenv.2020.110850 32531571 10.1016/j.ecoenv.2020.110850

[CR212] Yin D, Wang N, Xia F, Li Q, Wang W (2013) Impact of biocontrol agents *Pseudomonas fluorescens* 2P24 and CPF10 on the bacterial community in the cucumber rhizosphere. Eur J Soil Biol 59:36–42. 10.1016/j.ejsobi.2013.09.001

[CR178] Yoon I-H, Chang J-S, Lee J-H, Kim K-W (2009) Arsenite oxidation by *Alcaligenes* sp. strain RS-19 isolated from arsenic-contaminated mines in the Republic of Korea. Environ Geochem Health 31:109–117. 10.1007/s10653-008-9170-0 18642094 10.1007/s10653-008-9170-0

[CR179] Yu H, Leadbetter JR (2020) Bacterial chemolithoautotrophy via manganese oxidation. Nature 583:453–458. 10.1038/s41586-020-2468-5 32669693 10.1038/s41586-020-2468-5 PMC7802741

[CR180] Yu F, Lin J, Xie D, Yao Y, Wang X, Huang Y, Xin M, Yang F, Liu K, Li Y (2020) Soil properties and heavy metal concentrations affect the composition and diversity of the diazotrophs communities associated with different land use types in a mining area. Appl Soil Ecol 155:103669. 10.1016/j.apsoil.2020.103669

[CR181] Zargar K, Hoef S, Oremland R, Saltikov CW (2010) Identification of a novel arsenite oxidase gene, arxA, in the haloalkaliphilic, arsenite-oxidizing bacterium *Alkalilimnicola ehrlichii* strain MLHE-1. J Bacteriol 192(14):3755–3762. 10.1128/JB.00244-10 20453090 10.1128/JB.00244-10 PMC2897359

[CR182] Zawadzka AM, Crawford RL, Paszczynski AJ (2007) Pyridine-2,6-bis(thiocarboxylic acid) produced by *Pseudomonas stutzeri* KC reduces chromium(VI) and precipitates mercury, cadmium, lead and arsenic. Biometals 20:145–158. 10.1007/s10534-006-9022-2 16900399 10.1007/s10534-006-9022-2

[CR183] Zeroual Y, Moutaouakkil A, Blaghen M (2001) Volatilization of mercury by immobilized bacteria (*Klebsiella pneumoniae*) in different support by using fluidized bed bioreactor. Curr Microbiol 43:322–327. 10.1007/s002840010310 11688795 10.1007/s002840010310

[CR184] Zhang W, Chen L, Liu D (2012) Characterization of a marine-isolated mercury-resistant *Pseudomonas putida* strain SP1 and its potential application in marine mercury reduction. Appl Microbiol Biotechnol 93:1305–1314. 10.1007/s00253-011-3454-5 21751007 10.1007/s00253-011-3454-5

[CR185] Zhang J, Zhou W, Liu B, He J, Shen Q, Zhao F-J (2015) Anaerobic arsenite oxidation by an autotrophic arsenite-oxidizing *Bacterium* from an arsenic-contaminated paddy soil. Environ Sci Technol 49:5956–5964. 10.1021/es506097c 25905768 10.1021/es506097c

[CR186] Zhang Z, Yin N, Cai X, Wang Z, Cui Y (2016) Arsenic redox transformation by *Pseudomonas* sp. HN-2 isolated from arsenic-contaminated soil in Hunan. China. J Environ Sci 47:165–173. 10.1016/j.jes.2015.11.036 10.1016/j.jes.2015.11.036 27593283

[CR187] Zhang Y, Tang Y, Qin Z, Luo P, Ma Z, Tan M, Kang H, Huang Z (2019) A novel manganese oxidizing bacterium-*Aeromonas hydrophila* strain DS02: Mn(II) oxidization and biogenic Mn oxides generation. J Hazard Mat 367:539–545. 10.1016/j.jhazmat.2019.01.012 10.1016/j.jhazmat.2019.01.012 30654278

[CR188] Zhang R, Xu W, Shao S, Wang Q (2021) Gene silencing through CRISPR interference in bacteria: current advances and future prospects. Front Microbiol 12:635227. 10.3389/fmicb.2021.635227 33868193 10.3389/fmicb.2021.635227 PMC8044314

[CR189] Zhang W, Mao G, Zhuang J, Yang H (2023) The co-inoculation of Pseudomonas chlororaphis H1 and Bacillus altitudinis Y1 promoted soybean [Glycine max (L.) Merrill] growth and increased the relative abundance of beneficial microorganisms in rhizosphere and root. Front Microbiol 13:1079348. 10.3389/fmicb.2022.1079348 36699592 10.3389/fmicb.2022.1079348 PMC9868396

[CR190] Zhang Z, Zhang L, Zhang L, Chu H, Zhou J, Ju F (2024) Diversity and distribution of biosynthetic gene clusters in agricultural soil microbiomes. mSystems 9(4):e01263-23. 10.1128/msystems.01263-23 38470142 10.1128/msystems.01263-23 PMC11019929

[CR191] Zhao X, Do H, Zhou Y, Li Z, Zhang X, Zhao S, Li M, Wu D (2019) *Rahnella* sp. LRP3 induces phosphate precipitation of Cu (II) and its role in copper-contaminated soil remediation. J Hazard Mater 368:133–140. 10.1016/j.jhazmat.2019.01.029 30669037 10.1016/j.jhazmat.2019.01.029

[CR192] Zhao X, Teng Z, Wang G, Luo W, Guo Y, Ji X, Hu W, Li M (2023) Anaerobic syntrophic system composed of phosphate solubilizing bacteria and dissimilatory iron reducing bacteria induces cadmium immobilization via secondary mineralization. J Hazard Mater 446:130702. 10.1016/j.jhazmat.2022.130702 36587597 10.1016/j.jhazmat.2022.130702

[CR193] Zhu H, Teng Y, Wang X, Zhao L, Ren W, Luo Y, Christie P (2021) Changes in clover rhizosphere microbial community and diazotrophs in mercury-contaminated soils. Sci Total Environ 767:145473. 10.1016/j.scitotenv.2021.145473 33636759 10.1016/j.scitotenv.2021.145473

[CR194] Zuo Z, Gong T, Che Y, Liu R, Xu P, Jiang H, Yang C (2015) Engineering *Pseudomonas putida* KT2440 for simultaneous degradation of organophosphates and pyrethroids and its application in bioremediation of soil. Biodegrad 26:223–233. 10.1007/s10532-015-9729-2 10.1007/s10532-015-9729-2 25917649

