# Supplementary material for: Biotransformation of As, Cr, Hg, and Mn by Pseudomonadota: chances and risks
Source: Biodegradation. 2025 Jul 15;36(4):60. doi: 10.1007/s10532-025-10157-x (PMC12263811; doi:10.1007/s10532-025-10157-x)
Supplement: Supplementary file 1 — Supplementary file1 (PDF 481 KB) [file 10532_2025_10157_MOESM1_ESM.pdf]

S1 References for figure 1 (references in bold are both for fig. 1 and fig. 3)

1. Achal V., Pan X., Fu Q., Zhang D., 2012, Biomineralization based remediation of As(III) contaminated soil by Sporosarcina ginsengisoli, Journal of Hazardous Materials 201-202: 178-184; doi: 10.1016/j.jhazmat.2011.11.067
2. Achal V., Pan X., Lee D.-J., Kumari D., Zhang D., 2013, Remediation of Cr(VI) from chromium slag by biocementation, Chemosphere 93(7): 1352-1358; doi: 10.1016/j.chemosphere.2013.08.008
3. Achal V., Pan X., Zhang D., 2011, Remediation of copper-contaminated soil by Kocuria flava CR1, based on microbially induced calcite precipitation, Ecological Engineering 37(10): 1601-1605; doi: 10.1016/j.ecoleng.2011.06.008
4. Ahmad I., Akhtar M.J., Zahir Z.A., Naveed M., Mitter B., Sessitsch A., 2014, Cadmium-tolerant bacteria induce metal stress tolerance in cereals, Environ Sci Pollut Res 21: 11054-11065; doi: 10.1007/s11356-014-3010-9
5. **Ahmad W.A., Ahmad W.H.W., Karim N.A., Santhana Raj A.S., Zakaria Z.A., 2013, Cr(VI) reduction in naturally rich growth medium and sugarcane bagasse by Acinetobacter haemolyticus, International Biodeterioration & Biodegradation 85: 571e576; doi: 10.1016/j.ibiod.2013.01.008**
6. Al Disi Z., Attia E., Ahmad M.I., Zouari N., 2022, Immobilization of heavy metals by microbially induced carbonate precipitation using hydrocarbon-degrading ureolytic bacteria, Biotechnology Reports 35: e00747; doi: 10.1016/j.btre.2022.e00747
7. **Alam M.Z., Ahmad S., 2012, Toxic chromate reduction by resistant and sensitive bacteria isolated from tannery effluent contaminated soil, Ann Microbiol 62: 113-121; doi: 10.1007/s13213-011-0235-4**
8. Alam M.Z., Malik A., 2008, Chromate resistance, transport and bioreduction by Exiguobacterium sp. ZM-2 isolated from agricultural soil irrigated with tannery effluent, J Basic Microbiol 48: 416-420; doi: 10.1002/jobm.200800046
9. **An Q., Jin L., Deng S., Li Z., Zhang C., 2021, Removal of Mn(II) by a nitrifying bacterium Acinetobacter sp. AL-6: efficiency and mechanisms, Environmental Science and Pollution Research 28: 31218-31229; doi: 10.1007/s11356-021-12764-6**
10. **Andeer P.F., Learman D.R., McIlvin M., Dunn J.A., Hansel C.M., 2015, Extracellular haem peroxidases mediate Mn(II) oxidation in a marine Roseobacter bacterium via superoxide production, Environmental Microbiology 17(10): 3925-3936; doi: 10.1111/1462-2920.12893**
11. **Arshad M., Khan A.H.A., Hussain I., Badar-uz-Zamand, Anees M., Iqbal M., Soja G., Linde C., Yousaf S., 2017, The reduction of chromium (VI) phytotoxicity and phytoavailability to wheat (Triticum aestivum L.) using biochar and bacteria, Applied Soil Ecology 114: 90-98; doi: 10.1016/j.apsoil.2017.02.021**
12. **Bachate S.P., Khapare R.M., Kodam K.M., 2012, Oxidation of arsenite by two β-proteobacteria isolated from soil, Appl Microbiol Biotechnol 93: 2135-2145; doi: 10.1007/s00253-011-3606-7**
13. Bag P., Bhattacharya P., Chowdhury R., 2010, Bio-Detoxification of Arsenic Laden Ground Water Through a Packed Bed Column of a Continuous Flow Reactor Using Immobilized Cells, Soil and Sediment Contamination 19(4): 455-466; doi: 10.1080/15320383.2010.486050
14. **Bahar M.M., Megharaj M. Naidu R., 2016, Oxidation of arsenite to arsenate in growth medium and groundwater using a novel arsenite-oxidizing diazotrophic bacterium isolated from soil, International Biodeterioration & Biodegradation 106: 178-182; doi: 10.1016/j.ibiod.2015.10.019**

15. **Bahar Md.M., Megharaj M., Naidu R., 2012, Arsenic bioremediation potential of a new arsenite-oxidizing bacterium Stenotrophomonas sp. MM-7 isolated from soil, Biodegradation 23: 803-812; doi: 10.1007/s10532-012-9567-4**
16. Bai H., Liu D., Zheng W., Ma L., Yang S., Cao J., Lu X., Wang H., Mehta N., 2021, Microbially-induced calcium carbonate precipitation by a halophilic ureolytic bacterium and its potential for remediation of heavy metal-contaminated saline environments, International Biodeterioration & Biodegradation 165: 105311; doi: 10.1016/j.ibiod.2021.105311
17. **Baldi F., Parati F., Semplici, Tandoi V., 1993, Biological removal of inorganic Hg(II) as gaseous elemental Hg(0) by continuous culture of a Hg-resistant Pseudomonas puida strain FB-1, World Journal of Microbiology and Biotechnology 9: 275-279**
18. Banerjee A., Hazra A., Das S., Sengupta C., 2020, Groundwater inhabited Bacillus and Paenibacillus strains alleviate arsenic-induced phytotoxicity of rice plant, International Journal of Phytoremediation 22(10): 1048-1058; doi: 10.1080/15226514.2020.1725871
19. **Banerjee S., Datta S., Chattyopadhyay D., Sarkar P., 2011, Arsenic accumulating and transforming bacteria isolated from contaminated soil for potential use in bioremediation, Journal of Environmental Science and Health, Part A, 46(14): 1736-1747; doi: 10.1080/10934529.2011.623995**
20. Banerjee, S., Misra, A., Chaudhury, S., Dam, B., 2019, A Bacillus strain TCL isolated from Jharia coalmine with remarkable stress responses, chromium reduction capability and bioremediation potential, J. Hazard. Mater. 367: 215-223; doi: 10.1016/j.jhazmat.2018.12.038
21. **Batool K., Tuz Zahra F., Rehman Y., 2017, Arsenic-Redox Transformation and Plant Growth Promotion by Purple Nonsulfur Bacteria Rhodopseudomonas palustris CS2 and Rhodopseudomonas faecalis SS5, BioMed Research International 2017: 6250327; doi: 10.1155/2017/6250327**
22. **Batool R., Yrjälä K., Hasnain S., 2012, Hexavalent Chromium Reduction by Bacteria from Tannery J. Microbiol. Biotechnol. 22(4): 547-554; doi: 10.4014/jmb.1108.08029**
23. **Belzile N., Wu G.J., Chen Y.-W., Appanna V.D., 2006, Detoxification of selenite and mercury by reduction and mutual protection in the assimilation of both elements by Pseudomonas fluorescens, Science of the Total Environment 367: 704 – 714; doi: 10.1016/j.scitotenv.2006.03.008**
24. **Bhattacharya A., Gupta A., 2013, Evaluation of *Acinetobacter* sp. B9 for Cr (VI) resistance and detoxification with potential application in bioremediation of heavy-metals-rich industrial wastewater, Environ Sci Pollut Res 20: 6628–6637; doi: 10.1007/s11356-013-1728-4**
25. Bhattacharya A., Naik S.N., Khare S.K., 2018, Harnessing the bio-mineralization ability of urease producing Serratia marcescens and Enterobacter cloacae EMB19 for remediation of heavy metal cadmium (II), Journal of Environmental Management 215: 143-152; doi: 10.1016/j.jenvman.2018.03.055
26. Bilal S., Shahzad R., Khan A.L., Kang S.-M., Imran Q.M., Al-Harrasi A., Yun B.-W., Lee I.-J., 2019, Endophytic Microbial Consortia of Phytohormones-Producing Fungus Paecilomyces formosus LHL10 and Bacteria Sphingomonas sp. LK11 to Glycine max L. Regulates Physio-hormonal Changes to Attenuate Aluminum and Zinc Stresses, Front. Plant Sci. 9: 1273; doi: 10.3389/fpls.2018.01273
27. **Binish M.B., Shini S., Sinha R.K., Krishnan K.P., Mohan M., 2021, Mercuric reductase gene (merA) activity in a mercury tolerant sulphate reducing bacterium isolated from the Kongsfjorden, Arctic, Environ Sci Technol 38(1):104-11; doi: 10.1021/es034455a**
28. Bolan N., Kunhikrishnan A., Thangarajan R., Kumpiene J., Park J., Makino T., Kirkham M.B., Scheckel K., 2014, Remediation of heavy metal(loid)s contaminated soils – To mobilize or to immobilize?, Journal of Hazardous Materials 266: 141-166; doi: 10.1016/j.jhazmat.2013.12.018

29. Bomfield S.M., David D.J., 1976, Sorption and oxidation of manganous ions and reduction of manganese oxide by cell suspensions of a manganese oxidizing bacterium, Soil Biology and Biochemistry 8(1): 37-43

30. Bopp L.H., Ehrlich H.L., 1988, Chromate resistance and reduction in Pseudomonas fluorescens strain LB300, Arch Microbiol 150: 426-431; doi: 10.1007/BF00422281

31. Branco R., Francisco R., Chung A.P., Vasconcelos Morais P., 2009, Identification of an aox System That Requires Cytochrome c in the Highly Arsenic-Resistant Bacterium Ochrobactrum tritici SCII24, Appl Environ Microbiol 75(1): 5141–5147; doi: 10.1128/AEM.02798-08

32. Cai L., Rensing C., Li X., Wang G., 2004, Novel gene clusters involved in arsenite oxidation and resistance in two arsenite oxidizers: Achromobacter sp. SY8 and Pseudomonas sp. TS44, Applied Genetics and Molecular Biotechnology 83: 715-725; doi: 10.1007/s00253-009-1929-4

33. Cai Y., Yang K., Qiu C., Bi Y., Tian B., Bi X., 2023, A Review of Manganese-Oxidizing Bacteria (MnOB): Applications, Future Concerns, and Challenges, Int. J. Environ. Res. Public Health 20: 1272; doi: 10.3390/ijerph20021272

34. Calderón-Tovar I.L., Rietveld L.C., Araya-Obando J.A., Quesada-González A., Caballero-Chavarría A., Romero-Esquivel L.G., 2020, Autochthonous tropical groundwater bacteria involved in manganese(II) oxidation and removal, Environ. Sci.: Water Res. Technol. 6: 3132-3141; doi: 10.1039/d0ew00704h

35. Campos V.L., Valenzuela C., Yarza P., Kämpfer P., Vidal R., Zaror C., Mondaca M.-A., Lopez-Lopez A., Rosselló-Móra R., 2010, Pseudomonas arsenicoxydans sp nov., an arsenite-oxidizing strain isolated fromthe Atacama desert, Systematic and Applied Microbiology 33: 193–197; doi: 10.1016/j.syapm.2010.02.007

36. Campos-Guillén J., Caballero Pérez J., Cruz Medina J.A., Molina Vera C., Salas Rosas L.M., Limpens Gutiérrez C., García Salinas I., Hernández Ramírez M.R., Soto Alonso G., Cruz Hernández A., Saldaña Gutierrez C., Romero Gómez S., Pastrana Martínez X., Álvarez Hidalgo E., Gosar M., Dizdarevič T., 2014, Draft genome sequence of the mercury-resistant bacterium Acinetobacter idrijaensis strain MII, isolated from a mine-impacted area, Idrija, Slovenia, Genome Announc. 2(6):e01177-14; doi:10.1128/genomeA.01177-14

37. Casiot C., Morin G., Juillot F., Bruneel O., Personné J.-C., Leblanc M., Duquesne K., Bonnefoy V., Elbaz-Poulichet F., 2003, Bacterial immobilization and oxidation of arsenic in acid mine drainage (Carnoulès creek, France), Water Research 37(12): 2929-2936; doi: 10.1016/S0043-1354(03)00080-0

38. Chandhuru J., Harshitha S., Sujitha K., Mukesh Kumar D.J., 2012, Isolation of chromium resistant Bacillus sp. MRKV and reduction of hexavalent chromium potassium dichromate, J. Acad. Indus. Res. 1(6): 317-319

39. Chandraprabha M.N., Natarajan K.A., 2011, Mechanism of arsenic tolerance and bioremoval of arsenic by Acidithiobacilus ferrooxidans, Journal of Biochemical Technology 3(2): 257-265

40. Chang J.-S., Law W.-S., 1998, Development of microbial mercury detoxification processes using mercury-hyperresistant strain of Pseudomonas aeruginosa PU21, Biotechnology and Bioengineering 57(4): 462-470; doi: 10.1002/(SICI)1097-0290(19980220)57:4<462::AID-BIT10>3.0.CO;2-E

41. Chen J., Dong J., Shen S., Mei J., Chang J., 2019, Isolation of the Hg(II)-volatilizing Bacillus sp. strain DC-B2 and its potential to remediate Hg(II)-contaminated soils, 2019, J Chem Technol Biotechnol 94: 1433-1440; doi: 10.1002/jctb.5905

42. Cheng C., Wang Q., Wang Q.-X., He L.-Y., Sheng X.-F., 2021, Wheat-associated Pseudomonas taiwanensis WRS8 reduces cadmium uptake by increasing root surface cadmium adsorption and decreasing cadmium uptake and transport related gene expression in wheat, Environmental Pollution 268: 115850; doi: 10.1016/j.envpol.2020.115850

43. Cheung K.H., Lai H.Y., Gu J.-D., 2006, Membrane-Associated Hexavalent Chromium Reductase of Bacillus megaterium TKW3 with Induced Expression, J. Microbiol. Biotechnol. 16(6): 855-862
44. Chien M., Nakahata R., Ono T., Miyauchi K., Endo G., 2012, Mercury removal and recovery by immobilized Bacillus megaterium MB1, Front. Chem. Sci. Eng. 6(2): 192-197; doi: 10.1007/s11705-012-1284-3
45. Cooper D.C., Picardal F., Rivera J., Talbot C., 2000, Zinc Immobilization and Magnetite Formation via Ferric Oxide Reduction by Shewanella putrefaciens 200, Environ. Sci. Technol. 34(1): 100-106; doi: 10.1021/es990510x
46. Dary M., Chamber-Pérez M.A., Palomares A.J., Pajuelo E., 2009, "In situ" phytostabilisation of heavy metal polluted soils using Lupinus luteus inoculated with metal resistant plant-growth promoting rhizobacteria, J. Hazard. Mater., doi: 10.1016/j.jhazmat.2009.12.035
47. Das S., Jean J.-S., Chou M.-L., Rathod J., Liu C.-C., 2016, Arsenite-oxidizing bacteria exhibiting plant growth promoting traits isolated from the rhizosphere of Oryza sativa L.: Implications for mitigation of arsenic contamination in paddies, Journal of Hazardous Materials, 302: 10-18; doi: 10.1016/j.jhazmat.2015.09.044
48. Dash H.R., Mangwani N., Das S., 2014, Characterization and potential application in mercury bioremediation of highly mercury-resistant marine bacterium Bacillus thuringiensis PW-05, Environ Sci Pollut Res 21: 2642-2653; doi: 10.1007/s11356-013-2206-8
49. **Dash H.R., Sahu M., Mallick B., Das S., 2017, Functional efficiency of MerA protein among diverse mercury resistant bacteria for efficient use in bioremediation of inorganic mercury, Biochimie 142: 207-215; doi: 10.1016/j.biochi.2017.09.016**
50. **Dastidar A., Wang Y.-T., 2012, Modeling arsenite oxidation by chemoautotrophic Thiomonas arsenivorans strain b6 in a packed-bed bioreactor, Science of The Total Environment 432: 113-121; doi: 10.1016/j.scitotenv.2012.05.051**
51. **De J., Ramaiah N., Vardanyan L., 2008, Detoxification of Toxic Heavy Metals by Marine Bacteria Highly Resistant to Mercury, Mar Biotechnol 10: 471-477; doi: 10.1007/s10126-008-9083-z**
52. Duquesne K., Lebrun S., Casiot C., Bruneel O., Personné J.-C., Leblanc M., Elbaz-Poulichet F., Morin G., Bonnefoy V., 2003, Immobilization of Arsenite and Ferric Iron by Acidithiobacillus ferrooxidans and Its Relevance to Acid Mine Drainage, Applied and Environmental Microbiology 69(10): 6165-6173; doi: 10.1128/AEM.69.10.6165–6173.2003
53. **Dutta A., Mukherjee S.K., Hossain S.T., 2024, Characterization of As(III)-oxidizing bacteria *Acinetobacter* sp. TMKU7 having plant growth promoting features for possible application in arsenic-contaminated crop field, *Bioremediation Journal*, *28*(4), 457–471; doi: 10.1080/10889868.2023.2298326**
54. El-Meihy R.M., Abou-Alya H.E., Youssef A.M., Tewfike T.A., El-Alkshar E.A., 2019, Efficiency of heavy metals-tolerant plant growth promoting bacteria for alleviating heavy metals toxicity on sorghum, Environmental and Experimental Botany 162: 295-301; doi: 10.1016/j.envexpbot.2019.03.005
55. Essa A.M.M., Macaskie L.E., Brown N.L., 2005, A new method for mercury removal, Biotechnology Letters 27: 1649-1655; doi: 10.1007/s10529-005-2722-9
56. **Essahale A., Malki M., Marín I., Moumni M., 2012, Hexavalent Chromium Reduction and Accumulation by Acinetobacter AB1 Isolated from Fez Tanneries in Morocco, Indian J Microbiol 52(1): 48–53; doi: 10.1007/s12088-011-0187-1**
57. **Fan H., Su C., Wang Y., Yao J. , K. Zhao K., Wang Y., Wang G., 2008, Sedimentary arsenite-oxidizing and arsenate-reducing bacteria associated with high arsenic groundwater from Shanyin, Northwestern China, Journal of Applied Microbiology 105(2): 529–539; doi: 10.1111/j.1365-2672.2008.03790.x**

58. Fatnassi I.C., Chiboub M., Saadani O., Jebara M., Jebara S.H., 2015, Impact of dual inoculation with Rhizobium and PGPR on growth and antioxidant status of Vicia faba L. under copper stress, C. R. Biologies 338: 241-254; doi: 10.1016/j.crvi.2015.02.001

59. Ganguli A., Tripathi A.K., 1999, Survival and chromate reducing ability of Pseudomonas aeruginosa in industrial effluents, Letters in Applied Microbiology 28: 76–80; doi: 10.1046/j.1365-2672.1999.00457.x

60. Gao J., Wu S., Liu Y., Wu S., Jiang C., Li X., Wang R., Bai Z., Zhuang G., Zhuang X., 2020, Characterization and transcriptomic analysis of a highly Cr(VI)-resistant and -reductive plant-growth-promoting rhizobacterium Stenotrophomonas rhizophila DSM14405T, Environmental Pollution 263, Part B, 114622, doi:10.1016/j.envpol.2020.114622.

61. Garg S.K., Tripathi M., Singh S.K., Singh A., 2013, Pentachlorophenol dechlorination and simultaneous Cr6+ reduction by Pseudomonas putida SKG-1 MTCC (10510): characterization of PCP dechlorination products, bacterial structure, and functional groups, Environ Sci Pollut Res 20: 2288-2304; doi: 10.1007/s11356-012-1101-z

62. Ge S., Zhou M., Dong X., Lu Y., Ge S., 2013, Distinct and effective biotransformation of hexavalent chromium by a novel isolate under aerobic growth followed by facultative anaerobic incubation, Appl Microbiol Biotechnol 97: 2131-2137; doi: 10.1007/s00253-012-4361-0

63. Ghosh P., Rathinasabapathi B., Teplitski M., Ma L.Q., 2015, Bacterial ability in AsIII oxidation and AsV reduction: Relation to arsenic tolerance, P uptake, and siderophore production, Chemosphere 138: 995-1000; doi: 10.1016/j.chemosphere.2014.12.046

64. Gihring T.M., Banfield J.F., 2001, Arsenite oxidation and arsenate respiration by a new Thermus isolate, FEMS Microbiol Lett. 204(2): 335-40; doi: 10.1111/j.1574-6968.2001.tb10907.x

65. Giovanella P., Cabral L., Menezes Bento F., Gianello C., Oliveira Camargo F.A., 2016, Mercury (II) removal by resistant bacterial isolates and mercuric (II) reductase activity in a new strain of Pseudomonas sp. B50A, New Biotechnology 33(1): 216-223; doi: 10.1016/j.nbt.2015.05.006

66. Giovanella P., Cabral L., Pereira Costa A., de Oliveira Camargo F.A., Gianello C., Menezes Bento F., 2017, Metal resistance mechanisms in Gram-negative bacteria and their potential to remove Hg in the presence of other metals, Ecotoxicology and Environm. Safety 140: 162-169; doi: 10.1016/j.ecoenv.2017.02.010

67. Gomaa E.Z., 2019, Biosequestration of heavy metals by microbially induced calcite precipitation of ureolytic bacteria, Rom Biotechnol Lett. 24(1): 147-153; doi: 10.25083/rbl/24.1/147.153

68. González P.S., Ambrosio L.F., Paisio C.E., Talano M.A., Medina M.I., Agostini E., 2014, Chromium (VI) remediation by a native strain: effect of environmental conditions and removal mechanisms involved, Environ Sci Pollut Res 21: 13551-13559; doi: 10.1007/s11356-014-3311-z

69. Gounot A.-M., 1994, Microbial oxidation and reduction Consequences in groundwater and of manganese: applications, FEMS Microbiology Reviews 14: 339-350

70. Gupta S., Richa G., Jashan N., Swaranjit S.C., Nagaraja T., 2012, Biosequestration, Transformation, and Volatilization of Mercury by Lysinibacillus fusiformis Isolated from Industrial Effluent, J. Microbiol. Biotechnol. 22(5), 684–689, doi: 10.4014/jmb.1109.08022

71. Guria M.K., Guha A.K., Bhattacharya M., 2014, A green chemical approach for biotransformation of Cr(VI) to Cr(III), utilizing Fusarium sp. MMT1 and consequent structural alteration of cell morphology, Journal of Environmental Chemical Engineering 2: 424-433; doi: 10.1016/j.jece.2014.01.016

72. **Hamamura N., Fukushima K., Itai T., 2013, Identification of Antimony- and Arsenic-Oxidizing Bacteria Associated with Antimony Mine Tailing, Microbes Environ. 28(2): 257–263, doi:10.1264/jsme2.ME12217**
73. Han H., Wu X., Yao L., Chen Z., 2020, Heavy metal-immobilizing bacteria combined with calcium polypeptides reduced the uptake of Cd in wheat and shifted the rhizosphere bacterial communities, Environmental Pollution 267: 115432; doi: 10.1016/j.envpol.2020.115432
74. Han L.-J., Li J.-S., Chen Z., Xue Q., 2023, Stabilization of Pb(II) in wastewater and tailings by commercial bacteria through microbially induced phosphate precipitation (MIPP), Science of The Total Environment 868: 161628; doi: 10.1016/j.scitotenv.2023.161628
75. **He D., Zheng M., Ma T., Li C., Ni J., 2015, Interaction of Cr(VI) reduction and denitrification by strain Pseudomonas aeruginosa PCN-2 under aerobic conditions, Bioresource Technology 185: 346-352; doi: 10.1016/j.biortech.2015.02.109**
76. **He X., Xiao W., Zeng J., Tang J., Wang L., 2023, Detoxification and removal of arsenite by Pseudomonas sp. SMS11: Oxidation, biosorption and bioaccumulation, Bioresource Technology 185: 346–352; doi: 10.1016/j.biortech.2015.02.109**
77. He J., Chen X., Zhang Q., Achal V., 2019, More effective immobilization of divalent lead than hexavalent chromium through carbonate mineralization by Staphylococcus epidermidis HJ2, International Biodeterioration & Biodegradation 140: 67-71; doi: 10.1016/j.ibiod.2019.03.012
78. **He Z., Gao F., Sha T., Hu Y., He C., 2009, Isolation and characterization of a Cr(VI)-reduction Ochrobactrum sp. strain CSCr-3 from chromium landfill, J Hazard Mater 163: 869-873; doi: 10.1016/j.jhazmat.2008.07.041**
79. **Hosseinkhani B., Emtiazi G., 2011, Synthesis and Characterization of a Novel Extracellular Biogenic Manganese Oxide (Bixbyite-like Mn2O3) Nanoparticle by Isolated Acinetobacter sp., Curr Microbiol 63: 300–305; doi: 10.1007/s00284-011-9971-8**
80. **Hu L., Liu B., Li S., Zhong H., He Z., 2021, Study on the oxidative stress and transcriptional level in Cr(VI) and Hg(II) reducing strain *Acinetobacter indicus* yy-1 isolated from chromium-contaminated soil, Chemosphere 269: 128741; doi: 10.1016/j.chemosphere.2020.128741**
81. **Humphries A.C., Macaskie L.E., 2002, Reduction of Cr(VI) by Desulfovibrio vulgaris and Microbacterium, Biotechnology Letters 24: 1261-1267**
82. **Ibrahim A.S.S., Elbadawi Y.B., El-Tayeb M.A., Al-Salamah A.A., 2012b, Hexavalent chromium reduction by novel chromate resistant alkaliphilic Bacillus sp. strain KSUCr9a, African Journal of Biotechnology 11(16): 3832-3841; doi: 10.5897/AJB11.3026**
83. **Ibrahim A.S.S., El-Tayeb M.A., Elbadawi Y.B., Al-Salamah A.A., Antranikian G., 2012a, Hexavalent chromate reduction by alkaliphilic Amphibacillus sp. KSUCr3 is mediated by copper-dependent membrane-associated Cr(VI) reductase, Extremophiles 16: 659-668; doi: 10.1007/s00792-012-0464-x**
84. **Ilias M., Rafiqullah I.Md., Debnath B.C., Bin Mannan K.S., Hoq M.M., 2011, Isolation and Characterization of Chromium(VI)-Reducing Bacteria from Tannery Effluents, Indian J Microbiol 51(1): 76-81; doi: 10.1007/s12088-011-0095-4**
85. Islam F., Yasmeen T., Ali Q., Mubin M., Ali S., Arif M.S., Hussain S., Riaz M., Abbas F., 2015, Copper-resistant bacteria reduces oxidative stress and uptake of copper in lentil plants: potential for bacterial bioremediation, Environ Sci Pollut Res 23: 220-233; doi: 10.1007/s11356-015-5354-1
86. **Ito A., Miura J.-i., Ishikawa N., Umita T., 2012, Biological oxidation of arsenite in synthetic groundwater using immobilised bacteria, Water Research 46(15): 4825-4831; doi: 10.1016/j.watres.2012.06.013**
87. Jain D., Kour R., Bhojiya A.A., Meena R.H., Singh A., Mohanty S.R., Rajpurohit D.,Ameta K.D., 2020, Zinc tolerant plant growth promoting bacteria alleviates phytotoxic effects of zinc on

maize through zinc immobilization, Scientific Reports 10: 13865; doi: 10.1038/s41598-020-70846-w

88. Jalilvand N., Akhgar A., Alikhani H.A., Rahmani H.A., Rejali F., 2020, Removal of Heavy Metals Zinc, Lead, and Cadmium by Biomineralization of Urease-Producing Bacteria Isolated from Iranian Mine Calcareous Soils, Journal of Soil Science and Plant Nutrition 20: 206-219; doi: 10.1007/s42729-019-00121-z

89. Jiang N.-J., Liu R., Du Y.-J., Bi Y.-Z., 2019, Microbial induced carbonate precipitation for immobilizing Pb contaminants: Toxic effects on bacterial activity and immobilization efficiency, Science of The Total Environment 672: 722-731; doi: 10.1016/j.scitotenv.2019.03.294

90. Jong T., Parry D.L., 2004, Adsorption of Pb(II), Cu(II), Cd(II), Zn(II), Ni(II), Fe(II), and As(V) on bacterially produced metal sulfides, Journal of Colloid and Interface Science 275: 61-71; doi: 10.1016/j.jcis.2004.01.046

91. Kang C.-H., Han S.-H., Shin Y.J., Oh S.J., So J.-S., 2014, Bioremediation of Cd by Microbially Induced Calcite Precipitation, Appl Biochem Biotechnol 172: 2907-2915; doi: 10.1007/s12010-014-0737-1

92. Kang C.-H., Oh S.J., Shin Y., Han S.-H., Nam I.-H., So J.-S., 2015, Bioremediation of lead by ureolytic bacteria isolated from soil at abandoned metal mines in South Korea, Ecological Engineering 74: 402-407; doi: 10.1016/j.ecoenv.2023.114875

93. Karn S.K., Pan H., 2017, Bacterial Oxidation and Stabilization of As(III) in Soil, Environmental Engineering Science 34(3); doi: 10.1089/ees.2015.039

94. Karthik C, Barathi S, Pugazhendhi A, Ramkumar VS, Thi NBD, Arulselvi PI., 2017, Evaluation of Cr(VI) reduction mechanism and removal by Cellulosimicrobium funkei strain AR8, a novel haloalkaliphilic bacterium, J Hazard Mater 333:42-53, doi: 10.1016/j.jhazmat.2017.03.037

95. Katsoyiannis I.A., Zouboulis A.I., 2006, Use of Iron- and Manganese-Oxidizing Bacteria for the Combined Removal of Iron, Manganese and Arsenic from Contaminated Groundwater, Water Qual. Res. J. 41(2): 117-129; doi: 10.2166/wqrj.2006.014

96. Kavita B., Keharia H., 2012, Reduction of hexavalent chromium by Ochrobactrum intermedium BCR400 isolated from a chromium-contaminated soil, 3 Biotech 2(1): 79-87; doi: 10.1007/s13205-011-0038-0

97. Kitjanukit S., Takamatsu K., Okibe N., 2019, Natural Attenuation of Mn(II) in Metal Refinery Wastewater: Microbial Community Structure Analysis and Isolation of a New Mn(II)-Oxidizing Bacterium Pseudomonas sp. SK3, Water 11(3): 507; doi: 10.3390/w11030507

98. Kour R., Jain D., Bhojiya A.A., Sukhwal A., Sanadhya S., Saheewala H., Jat G., Singh A., Mohanty S.R., 2019, Zinc biosorption, biochemical and molecular characterization of plant growth‑promoting zinc‑tolerant bacteria, 3 Biotech 9: 421; doi: 10.1007/s13205-019-1959-2

99. Kozieł M., 2022, Properties, various functions and application of bacterial melanins – some properties and possible application of melanin produced by Azotobacter chroococcum, Polish Journal of Agronomy 51: 46-54; doi: 10.26114/pja.iung.498.2022.51.04

100. Krumholz L.R., Elias D.A., Suflita J.M., 2003, Immobilization of Cobalt by Sulfate-Reducing Bacteria in Subsurface Sediments, Geomicrobiology Journal 20: 61-72; doi: 10.1080/01490450390144376

101. Kuffner M., De Maria S., Puschenreiter M., Fallmann K., Wieshammer G., Gorfer M., Strauss J., Rivelli A.R, Sessitsch A., 2010, Culturable bacteria from Zn- and Cd-accumulating Salix caprea with differential effects on plant growth and heavy metal availability, Journal of Applied Microbiology 108(4): 1471-1484; doi: 10.1111/j.1365-2672.2010.04670.x

102. Kuffner M., Puschenreiter M., Wieshammer G., Gorfer M., Sessitsch A., 2008, Rhizosphere bacteria affect growth and metal uptake of heavy metal accumulating willows, Plant Soil 304: 35-44; doi: 10.1007/s11104-007-9517-9

103. Kumari D., Pan X., Lee D.-J., Achal V., 2014, Immobilization of cadmium in soil by microbially induced carbonate precipitation with Exiguobacterium undae at low temperature, International Biodeterioration & Biodegradation 94: 98-102; doi: 10.1016/j.ibiod.2014.07.007

104. Lakshmanan V., Shantharaj D., Li G., Seyfferth A.L., Sherrier D.J., Bais H.P., 2015, A natural rice rhizospheric bacterium abates arsenic accumulation in rice (Oryza sativa L.), Planta 242: 1037-1050; doi: 10.1007/s00425-015-2340-2

105. Li W., Fishman A., Achal V., 2021, Ureolytic bacteria from electronic waste area, their biological robustness against potentially toxic elements and underlying mechanisms, Journal of Environmental Management, 289: 112517; doi: 10.1016/j.jenvman.2021.112517

106. Li W., Yang Y., Achal V., 2022 a, Biochemical composite material using corncob powder as a carrier material for ureolytic bacteria in soil cadmium immobilization, Science of The Total Environment 802: 149802; doi: 10.1016/j.scitotenv.2021.149802

107. **Li Y., Guo L., Häggblom M.M., Yang R., Li M., Sun X., Chen Z., Li F., Su X., Yan G., Xiao E., Zhang H., Sun W., 2022 b, Serratia spp. Are Responsible for Nitrogen Fixation Fueled by As(III) Oxidation, a Novel Biogeochemical Process Identified in Mine Tailings, Environmental Science & Technology 56(3): 2033-2043; doi: 10.1021/acs.est.1c06857**

108. Liao S., Zhou J., Wang H., Chen X., Wang H., Wang G., 2013, Arsenite Oxidation Using Biogenic Manganese Oxides Produced by a Deep-Sea Manganese-Oxidizing Bacterium, Marinobacter sp. MnI7-9, Geomicrobiology Journal 30(2): 150-159; doi: 10.1080/01490451.2011.654379

109. Liao V.H.-C., Chu Y.-J., Su Y.-C., Hsiao S.-Y., Wei C.-C., Liu C.-W., Liao C.-M., Shen W.-C., Chang F.-J., 2011, Arsenite-oxidizing and arsenate-reducing bacteria associated witharsenic-rich groundwater in Taiwan, Journal of Contaminant Hydrology 123(1–2): 20-29; doi: 10.1016/j.jconhyd.2010.12.003

110. Liu P., Zhang Y., Tang Q., Shi S., 2021, Bioremediation of metal-contaminated soils by microbially-induced carbonate precipitation and its effects on ecotoxicity and long-term stability, Biochemical Engineering Journal 166: 107856; doi: 10.1016/j.bej.2020.107856

111. Liu Y., Serrano A., Wyman V., Marcellin E., Southam G., Vaughan J., Villa-Gomez D., 2021, Nickel complexation as an innovative approach for nickel-cobalt selective recovery using sulfate-reducing bacteria, Journal of Hazardous Materials 402: 123506; doi: 10.1016/j.jhazmat.2020.123506

112. Lu X., Zhang Y., Liu C., Wu M., Wang H., 2018, Characterization of the antimonite- and arsenite-oxidizing bacterium Bosea sp. AS-1 and its potential application in arsenic removal, Journal of Hazardous Materials, 359: 527-534; doi: 10.1016/j.jhazmat.2018.07.112

113. Ma Y., Zhong H., He Z., 2019, Cr(VI) reductase activity locates in the cytoplasm of Aeribacillus pallidus BK1, a novel Cr(VI)-reducing thermophile isolated from Tengchong geothermal region, China, Chemical Engineering Journal 371: 524-534 doi: 10.1016/j.cej.2019.04.085.

114. Macur R.E., Jackson C.R., Botero L.M., McDermott T.R., Inskeep W.P., 2004, Bacterial Populations Associated with the Oxidation and Reduction of Arsenic in an Unsaturated Soil, Environ. Sci. Technol. 38: 104-111; doi: 10.1021/es034455a

115. Madhaiyan M., Poonguzhali S., Sa T., 2007, Metal tolerating methylotrophic bacteria reduces nickel and cadmium toxicity and promotes plant growth of tomato (Lycopersicon esculentum L.), Chemosphere 69(2): 220-228; doi: 10.1016/j.chemosphere.2007.04.017

116. Mahbub K.R., Krishnan K., Megharaj M., Naidu R., 2016a, Bioremediation potential of a highly mercury resistant bacterial strain Sphingobium SA2 isolated from contaminated soil, Chemosphere 144: 330-337; doi: 10.1016/j.chemosphere.2015.08.061

117. Mahbub K.R., Krishnan K., Naidu R., Megharaj M., 2016b, Mercury resistance and volatilization by Pseudoxanthomonas sp. SE1 isolated from soil, Environmental Technology & Innovation 6: 94-104; doi: 10.1016/j.eti.2016.08.001

118. Mallick I., Bhattacharyya C., Mukherji S., Dey D., Sarkar S.C., Mukhopadhyay U.K., Ghosh A., 2018, Effective rhizoinoculation and biofilmformation by arsenic immobilizing halophilic plant growth promoting bacteria (PGPB) isolated from mangrove rhizosphere: A step towards arsenic rhizoremediation, Science of the Total Environment 610–611: 1239-1250; doi: 10.1016/j.scitotenv.2017.07.234

119. Mao Q., Wei D., Yan B., Luo S., Seviour T.W., Wei Z., Xie X., Luo L., 2022, Removal of manganese in acidic solutions utilizing *Achromobacter* sp. strain QBM-4 isolated from mine drainage Process Safety and Environmental Protection 165: 8513-8522; doi: 10.1016/j.psep.2022.04.002

120. Martinez R.E., Pedersen K., Ferris F.G., 2004, Cadmium complexation by bacteriogenic iron oxides from a subterranean environment, Journal of Colloid and Interface Science 275: 82-89; doi: 10.1016/j.jcis.2004.02.018

121. Martins M., Faleiro M.L., Barros R.J., Veríssimo A.R., Barreiros M.A., Costa M.C., 2009, Characterization and activity studies of highly heavy metal resistant sulphate-reducing bacteria to be used in acid mine drainage decontamination, Journal of Hazardous Materials 166: 706-713; doi: 10.1016/j.jhazmat.2008.11.088

122. Marzban A., Ebrahimipour G., Karkhane M., Teymouri M., 2016, Metal resistant and phosphate solubilizing bacterium improves maize (Zea mays) growth and mitigates metal accumulation in plant, Biocatalysis and Agricultural Biotechnology 8: 13-17; doi: 10.1016/j.bcab.2016.07.005

123. Miranda-Carrazco, A., Vigueras-Cortés, J.M., Villa-Tanaca, L. Hernandez-Rodriguez C., 2018, Cyanotrophic and arsenic oxidizing activities of Pseudomonas mendocina P6115 isolated from mine tailings containing high cyanide concentration. Arch Microbiol 200: 1037–1048; doi: 10.1007/s00203-018-1514-2

124. Mohr J.F., Baldeweg F., Deicke M., Morales Reyes C.F., Hoffmeister D., Wichard T., 2021, Frankobactin Metallophores Produced by Nitrogen-Fixing Frankia Actinobacteria Function in Toxic Metal Sequestration, J. Nat. Prod., 84(4): 1216-1225; doi: 10.1021/acs.jnatprod.0c01291

125. Mondaca M.A., Campos V., Moraga R., Zaror C.A., 2002, Chromate Reduction in Serratia marcescens Isolated from Tannery Effluent and Potential Application for Bioremediation of Chromate Pollution, The Scientific World JOURNAL 2: 972-977; doi: 10.1100/tsw.2002.154

126. Mugwar A., Harbottle M.J., 2016, Toxicity effects on metal sequestration by microbially-induced carbonate precipitation, Journal of Hazardous Materials 314: 237-248; doi: 10.1016/j.jhazmat.2016.04.039

127. Mwandira W., Nakashima K., Kawasaki S., 2017, Bioremediation of lead-contaminated mine waste by Pararhodobacter sp. based on the microbially induced calcium carbonate precipitation technique and its effects on strength of coarse and fine grained sand, Ecol. Eng. 109(A): 57-64; doi: 10.1016/j.ecoleng.2017.09.011

128. Nakamura K., Aoki J., Morishita K., Yamamoto M., 2000, Mercury volatilization by the most mercury-resistant bacteria from the seawater of Minamata Bay in various physiological conditions, Clean Products and Processes 2: 174-178; doi: 10.1007/s100980000079

129. Narayani M., Vidya Shetty K., 2012, Characteristics of a Novel Acinetobacter sp. and Its Kinetics in Hexavalent Chromium Bioreduction, J. Microbiol. Biotechnol. 22(5): 690-698; doi: 10.4014/jmb.1110.10073

130. Nguema P.F., Luo Z., 2012, Aerobic chromium(VI) reduction by chromium-resistant bacteria isolated from activated sludge, Ann Microbiol 62: 41-47; doi: 10.1007/s13213-011-0224-7

131. Nguyen V.K., Ha M.-G., Kang H.Y., Nguyen D.D., 2020, Biological Manganese Removal by Novel Halotolerant Bacteria Isolated from River Water, Biomolecules 10: 941; doi: 10.3390/biom10060941

132. Nookongbut P., Kantachote D., Megharaj M., Naidu R., 2018, Reduction in arsenic toxicity and uptake in rice (Oryza sativa L.) by As-resistant purple nonsulfur bacteria, Environmental Science and Pollution Research 25: 36530-36544; doi: 10.1007/s11356-018-3568-8

133. Okino S., Iwasaki K., Yagi O., Tanaka H., 2000, Development of a biological mercury removal-recovery system, Biotechnology Letters 22: 783-788

134. Opperman D.J., van Heerden E., 2007, Aerobic Cr(VI) reduction by Thermus scotoductus strain SA-01, J Appl Microbiol 103: 1097-1913; doi: 10.1111/j.1365-2672.2007.03429.x

135. Ozturk S., Kaya T., Aslim B., Tan S., 2012, Removal and reduction of chromium by Pseudomonas spp. and their correlation to rhamnolipid production, Journal of Hazardous Materials 231–232: 64–69; doi: 10.1016/j.jhazmat.2012.06.038

136. Pal A., Dutta S., Paul A.K., 2005, Reduction of Hexavalent Chromium by Cell-Free Extract of Bacillus sphaericus AND 303 Isolated from Serpentine Soil, Current microbiology 51: 327-330; doi: 10.1007/s00284-005-0048-4

137. Park J.H., Bolan N., Megharaj M., Naidua R., 2011, Isolation of phosphate solubilizing bacteria and their potential for lead immobilization in soil, Journal of Hazardous Materials 185: 829-836; doi: 10.1016/j.jhazmat.2010.09.095

138. Pepi M., Focardi S., Tarabelli A., Volterrani M., Focardi S.E., 2013, Bacterial strains resistant to inorganic and organic forms of mercury isolated from polluted sediments of the Orbetello Lagoon, Italy, and their possible use in bioremediation processes, E3S Web of Conferences 1: 31002; doi: 10.1051/e3sconf/20130131002

139. Piazza A., Ciancio Casalini L., Pacini V.A., Sanguinetti G., Ottado J., Gottig N., 2019, Environmental Bacteria Involved in Manganese(II) Oxidation and Removal From Groundwater, Front. Microbiol. 10: 119; doi: 10.3389/fmicb.2019.00119

140. Pishchik V.N., Vorobyev N.I., Chernyaeva I.I., Timofeeva S.V., Kozhemyakov A.P., Alexeev Y.V., Lukin S.M., 2002, Experimental and mathematical simulation of plant growth promoting rhizobacteria and plant interaction under cadmium stress, Plant and Soil 243: 173-186

141. Prabhakaran D.C., Bolaños-Benitez V., Sivry Y., Gelabert A., Riotte J., Subramanian S., 2019, Mechanistic studies on the bioremediation of Cr(VI) using Sphingopyxis macrogoltabida SUK2c, a Cr(VI) tolerant bacterial isolate, Biochemical Engineering Journal 150: 107292; doi: 10.1016/j.bej.2019.107292

142. Prabhakaran D.C., Subramanian S., 2017, Studies on the Bioremediation of Chromium from Aqueous Solutions Using C. paurometabolum, Trans Indian Inst Met 70(2): 497–509; doi: 10.1007/s12666-016-1009-2

143. Pramanik K., Mandal S., Banerjee S., Ghosh A., Maiti T.K., Mandal N.C., 2021, Unraveling the heavy metal resistance and biocontrol potential of Pseudomonas sp. K32 strain facilitating rice seedling growth under Cd stress, Chemosphere 274: 129819; doi: 10.1016/j.chemosphere.2021.129819

144. Princy S., Sathish S.S., Cibichakravarthy B., Prabagaran S.R., 2020, Hexavalent chromium reduction by Morganella morganii (1Ab1) isolated from tannery effluent contaminated sites of Tamil Nadu, India, Biocatalysis and Agricultural Biotechnology 23: 101469, doi: 10.1016/j.bcab.2019.101469

145. Pulimi M., Jamwal S., Samuel J., Chandrasekaran N., Mukherjee N., 2012, Enhancing the Hexavalent Chromium Bioremediation Potential of Acinetobacter junii VITSUKMW2 Using Statistical Design Experiments, J. Microbiol. Biotechnol. 22(12): 1767–1775, doi: 10.4014/jmb.1203.03063

146. Qiao S., Zeng G., Wang X., Dai C., Sheng M., Chen Q., Xu F., Xu H., 2021, Multiple heavy metals immobilization based on microbially induced carbonate precipitation by ureolytic bacteria and the precipitation patterns exploration, Chemosphere 274: 129661; doi: 10.1016/j.chemosphere.2021.129661
147. Qin S., Wu X., Han H., Pang F., Zhang J., Chen Z., 2021, Polyamine-producing bacterium Bacillus megaterium N3 reduced Cd accumulation in wheat and increased the expression of DNA repair- and plant hormone- related proteins in wheat roots, Environmental and Experimental Botany 189: 104563; doi: 10.1016/j.envexpbot.2021.104563
148. Qin S., Zhang H., He Y., Chen Z., Yao L., Han H., 2023, Improving radish phosphorus utilization efficiency and inhibiting Cd and Pb uptake by using heavy metal-immobilizing and phosphate-solubilizing bacteria, Science of The Total Environment 868: 161685; doi: 10.1016/j.scitotenv.2023.161685
149. Queiroz P.S., Barboza N.R., Cordeiro M.M., Leão V.A., Guerra-Sá R., 2018, Rich growth medium promotes an increased on Mn(II) removal and manganese oxide production by Serratia marcescens strains isolates from wastewater, Biochemical Engineering Journal 140: 148-156; doi: 10.1016/j.bej.2018.09.018
150. Ram Talib N.S., Effendi Halmi M.I., Abd Ghani S.S., Zaidan U.H., Abd Shukor M.Y., 2019, Artificial Neural Networks (ANNs) and Response Surface Methodology (RSM) Approach for Modelling the Optimization of Chromium (VI) Reduction by Newly Isolated Acinetobacter radioresistens Strain NS-MIE from Agricultural Soil, BioMed Research International 2019: 5785387; doi: 10.1155/2019/5785387
151. Rajasabapathy R., Mohandass C., Dastager S.G., Liu Q., Li W.-J., Colaço A., 2015, Citreicella manganoxidans sp. nov., a novel manganese oxidizing bacterium isolated from a shallow water hydrothermal vent in Espalamaca (Azores), Antonie van Leeuwenhoek 108: 1433-1439; doi: 10.1007/s10482-015-0597-x
152. Ravanbakhsh M., Kowalchuk G.A., Jousset A., 2019, Optimization of plant hormonal balance by microorganisms prevents plant heavy metal accumulation, Journal of Hazardous Materials 379: 120787; doi: 10.1016/j.jhazmat.2019.120787
153. Rehman A., Zahoor A., Muneer B., Hasnain S., 2008, Chromium Tolerance and Reduction Potential of a Bacillus sp.ev3 Isolated from Metal Contaminated Wastewater, Bull Environ Contam Toxicol 81: 25-29; doi: 10.1007/s00128-008-9442-5
154. Rizvi A., Khan M.S., 2018, Heavy metal induced oxidative damage and root morphology alterations of maize (Zea mays L.) plants and stress mitigation by metal tolerant nitrogen fixing Azotobacter chroococcum, Ecotoxicology and Environmental Safety 157: 9-20; doi: 10.1016/j.ecoenv.2018.03.063
155. Rodríguez-Sánchez V., Guzmán-Moreno J., Rodríguez-González V., Flores-de la Torre J.A., Ramírez-Santoyo R.M., Vidales-Rodríguez L.E., 2017, Biosorption of lead phosphates by lead-tolerant bacteria as a mechanism for lead immobilization, World Journal of Microbiology and Biotechnology 33: 150; doi: 10.1007/s11274-017-2314-6
156. Sagar S., Dwivedi A., Yadav S., Tripathi M., Kaistha S.D., 2012, Hexavalent chromium reduction and plant growth promotion by Staphylococcus arlettae strain Cr11, Chemosphere 86: 847-852; doi: 10.1016/j.chemosphere.2011.11.031
157. Salmassi T.M., Venkateswaren K., Satomi M., Newman D.K., Hering J.G., 2002, Oxidation of Arsenite by Agrobacterium albertimagni, AOL15, sp. nov., Isolated from Hot Creek, California, Geomicrobiology Journal 19(1): 53-66; doi: 10.1080/014904502317246165
158. Samantaray D.P., Mishra B.B., 2012, Effect of metal on hexavalent chromium reduction by Acinetobacter calcoaceticus, The Bioscan 7(4): 627-629

159. Sandana Mala J.G., Sujatha D., Rose C., 2015, Inducible chromate reductase exhibiting extracellular activity inBacillus methylotrophicus for chromium bioremediation, Microbiological Research 170: 235-241; doi: 10.1016/j.micres.2014.06.001

160. Sanjay M.S., Sudarsanam D., Raj G.A., Baskar K., 2020, Isolation and identification of chromium reducing bacteria from tannery effluent, Journal of King Saud University - Science 32(1): 265-271, doi: 10.1016/j.jksus.2018.05.001.Saran et al. 2020 [270]

161. Saran A., Imperato V., Fernandez L., Gkorezis P., d'Haen J., Merini L.J., Vangronsveld J., Thijs S., 2020, Phytostabilization of Polluted Military Soil Supported by Bioaugmentation with PGP-Trace Element Tolerant Bacteria Isolated from Helianthus petiolaris, Agronomy 10(2): 204; doi: 10.3390/agronomy10020204

162. Sarangi A., Krishnan C., 2008, Comparison of in vitro Cr(VI) reduction by CFEs of chromate resistant bacteria isolated from chromate contaminated soil, Bioresource Technology 99: 4130-4137; doi: 10.1016/j.biortech.2007.08.059

163. Sathishkumar K., Murugan K., Benelli G., Higuchi A., Rajasekar A., 2016, Bioreduction of hexavalent chromium by Pseudomonas stutzeri L1 and Acinetobacter baumannii L2, Ann Microbiol 67: 91–98; doi: 10.1007/s13213-016-1240-4

164. Sayel H., Bahafid W., Joutey N.T., Derraz K., Benbrahim K.F., Koraichi S.I., El Ghachtouli N., 2012, Cr(VI) reduction by Enterococcus gallinarum isolated from tannery waste-contaminated soil, Ann Microbiol 62: 1269-1277; doi: 10.1007/s13213-011-0372-9

165. Sevak P., Pushkar B., Mazumdar S., 2023, Mechanistic evaluation of chromium bioremediation in *Acinetobacter junii* strain b2w: A proteomic approach, Journal of Environmental Management 328: 116978; doi: 10.1016/j.jenvman.2022.116978

166. Shen S., Li Y., Chen M., Huang J., Liu F., Xie S., Kong L., Pan Y., Reduced cadmium toxicity in rapeseed via alteration of root properties and accelerated plant growth by a nitrogen-fixing bacterium, Journal of Hazardous Materials 449: 131040; doi: 10.1016/j.jhazmat.2023.131040

167. Shi Y., Chai L., Yang Z., Jing Q., Chen R., Chen Y., 2012, Identification and hexavalent chromium reduction characteristics of Pannonibacter phragmitetus, Bioprocess Biosyst Eng 35: 843-850; doi: 10.1007/s00449-011-0668-y

168. Shi Y., Wang Z., Li H., Yan Z., Meng Z., Liu C., Chen J., Duan C., 2023, Resistance mechanisms and remediation potential of hexavalent chromium in Pseudomonas sp. strain AN-B15, Ecotoxicology and Environmental Safety 250: 114498; doi: 10.1016/j.ecoenv.2023.114498

169. Silva B., Figueiredo H., Quintelas C., Neves I.C., Tavares T., 2012, Improved biosorption for Cr(VI) reduction and removal by Arthrobacter viscosus using zeolite, International Biodeterioration & Biodegradation 74: 116-123; doi: 10.1016/j.ibiod.2012.05.026

170. Singh A.L., Singh V.K., Yadav A., 2016, Arsenic sequestration by manganese-oxidizing Acinetobacter sp., Indian Journal of Biotechnology 15: 525-530

171. Sinha S., Mukherjee S.K., 2008, Cadmium–Induced Siderophore Production by a High Cd-Resistant Bacterial Strain Relieved Cd Toxicity in Plants Through Root Colonization, Curr Microbiol 56: 55-60; doi: 10.1007/s00284-007-9038-z

172. Smrithi A., Usha K., 2012, Isolation and characterization of chromium removing bacteria from tannery effluent disposal site, International Journal of Advanced Biotechnology and Research 3(3): 644-652

173. Soni S.K., Singh R., Awasthi A., Singh M., Kalra A., 2013, In vitro Cr(VI) reduction by cell-free extracts of chromate-reducing bacteria isolated from tannery effluent irrigated soil, Environ Sci Pollut Res 20: 1661-1674; doi: 10.1007/s11356-012-1178-4

174. Subramanian S., Sam S., Jayaraman G., 2012, Hexavalent chromium reduction by metal resistant and halotolerant Planococcus maritimus VITP21, African Journal of Microbiology Research 6(47): 7339-7349; doi: 10.5897/AJMR12.143

175. Tan H., Wang C., Zeng G., Luo Y., Li H., Xu H., 2020, Bioreduction and biosorption of Cr(VI) by a novel Bacillus sp. CRB-B1 strain, Journal of Hazardous Materials 386: 121628; doi: 10.1016/j.jhazmat.2019.121628
176. Tang W.W., Gong J.M., Wu L.J., Li Y.F., Zhang M.T., Zeng X.P., 2016, DGGE diversity of Mn mine samples and isolation of a Lysinibacillus sp. efficient in removal of high Mn (II) concentrations, Chemosphere 165: 277-283; doi: 10.1016/j.chemosphere.2016.08.134
177. Tang W.W., Xia J., Zeng X.P., Wu L.J., Ye G.Q., 2014, Biological characteristics and oxidation mechanism of a new Mn-oxidizing bacteria FM-2. Bio-Med. Mater. Eng. 24: 703-709; doi: 10.3233/BME-130858
178. Templeton A.S., Trainor T.P., Spormann A.M., Newville M., Sutton S.R., Dohnalkova A., Gorby Y., Brown G.E.Jr, 2003, Sorption versus Biomineralization of Pb(II) within Burkholderia cepacia Biofilms, Environ. Sci. Technol. 37(2): 300-307; doi: 10.1021/es025972g
179. Teng Z., Shao W., Zhang K., Huo Y., Li M., 2019, Characterization of phosphate solubilizing bacteria isolated from heavy metal contaminated soils and their potential for lead immobilization, Journal of Environmental Management 231: 189-197; doi: 10.1016/j.jenvman.2018.10.012
180. Thacker U., Parikh R., Shouche Y., Madamwar D., 2007, Reduction of chromate by cell-free extract of Brucella sp. isolated from Cr(VI) contaminated sites, Bioresource Technology 98: 1541-1547; doi: 10.1016/j.biortech.2006.06.011
181. Tharannum S., Krishnamurthy V., Mahmood R., 2012, Characterization of chromium remediating bacterium Bacillus subtilis isolated from electroplating effluent, International Journal of Engineering Research and Applications 2(4): 961-966
182. Toyoda K., Tebo B.M., 2013, The effect of Ca2+ ions and ionic strength on Mn (II) oxidation by spores of the marine Bacillus sp. SG-1, Geochim. Cosmochim. Acta 101: 1-11; doi: 10.1016/j.gca.2012.10.008
183. Upadhyay S., Tarafdar A., Sinha A., 2018, Assessment of Serratia sp. isolated from Iron Ore mine in Hexavalent Chromium reduction: Kinetics, Fate and Variation in Cellular Morphology, Environmental Technology; doi: 10.1080/09593330.2018.1521875
184. Viamajala S., Smith W.A., Sani R.K., Apel W.A., Petersen J.N., Neal A.L., Roberto F.F., Newby D.T., Peyton B.M., 2007, Isolation and characterization of Cr(VI) reducing Cellulomonas spp. from subsurface soils: Implications for long-term chromate reduction, Bioresource Technology 98: 612-622; doi: 10.1016/j.biortech.2006.02.023
185. Vivas A., Biró B., Ruíz-Lozano J.M., Barea J.M., Azcón R., 2006, Two bacterial strains isolated from a Zn-polluted soil enhance plant growth and mycorrhizal efficiency under Zn-toxicity, Chemosphere 62(9): 1523-1533; doi: 10.1016/j.chemosphere.2005.06.053
186. Von Canstein H., Kelly S., Li Y., Wagner-Döbler I., 2002, Species Diversity Improves the Efficiency of Mercury-Reducing Biofilms under Changing Environmental Conditions, Appl. Environ. Microbiol. 68(6): 2829-2837; doi: 10.1128/AEM.68.6.2829–2837.2002
187. Von Canstein H., Li Y., Timmis K.N., Deckwer W.-D.,Wagner-Döbler I., 1999, Removal of Mercury from Chloralkali Electrolysis Wastewater by a Mercury-Resistant Pseudomonas putida Strain, Applied and Environmental Microbiology 65(12): 5279-5284
188. Wagner- Döbler I., von Canstein H., Li Y., Timmis K.N., Deckwer W.-D., 2000, Removal of Mercury from Chemical Wastewater by Microoganisms in Technical Scale, Environ. Sci. Technol. 34(21): 4628-4634; doi: 10.1021/es0000652
189. Wan W., Qin Y., Wu H., Zuo W., He H., Tan J., Wang Y., He D., 2020, Isolation and Characterization of Phosphorus Solubilizing Bacteria With Multiple Phosphorus Sources Utilizing Capability and Their Potential for Lead Immobilization in Soil, Front. Microbiol. 11: 752; doi: 10.3389/fmicb.2020.00752

190. Wang K.T., Li Y.P., Wu Y.C., Qiu Z.Q., Ding Z.X., Wang X.J., Chen W., Wang R.J., Fu F.F., Rensing C., Yang G.D., 2020b, Improved grain yield and lowered arsenic accumulation in rice plants by inoculation with arsenite-oxidizing Achromobacter xylosoxidans GD03, Ecotoxicology and Environmental Safety 206: 111229; doi: 10.1016/j.ecoenv.2020.111229

191. Wang T., Wang S., Tang X., Fan X., Yang S., Yao L., Li Y., Han H., 2020c, Isolation of urease-producing bacteria and their effects on reducing Cd and Pb accumulation in lettuce (Lactuca sativa L.), Environmental Science and Pollution Research 27: 8707-8718; doi: 10.1007/s11356-019-06957-3

192. Wang W., Shao Z., Liu Y., Wang G., 2009, Removal of multi-heavy metals using biogenic manganese oxides generated by a deep-sea sedimentary bacterium – Brachybacterium sp. strain Mn32, Microbiology 155: 1989-1996; doi: 10.1099/mic.0.024141-0

193. Wang X., Yu M., Wang L., Lin H., Li B., Xue C.-X., Sun H., Zhang X.-H., 2020a, Comparative genomic and metabolic analysis of manganese-oxidizing mechanisms in Celeribacter manganoxidans DY25T: Its adaptation to the environment of polymetallic nodules, Genomics 112: 2080-2091; doi: 10.1016/j.ygeno.2019.12.002

194. Wang X.-H., Luo W.-W., Wang Q., He L.-Y., Sheng X.-F., 2018, Metal(loid)-resistant bacteria reduce wheat Cd and As uptake in metal(loid)-contaminated soil, Environmental Pollution 241: 529-539; doi: 10.1016/j.envpol.2018.05.088

195. Wani P.A., Khan M.S., Zaidi A., 2007, Effect of metal tolerant plant growth promoting Bradyrhizobium sp. (vigna) on growth, symbiosis, seed yield and metal uptake by greengram plants, Chemosphere 70(1): 36-45; doi: 10.1016/j.chemosphere.2007.07.028

196. Wani P.A., Khan M.S., Zaidi A., 2008, Effect of Metal-Tolerant Plant Growth-Promoting Rhizobium on the Performance of Pea Grown in Metal-Amended Soil, Arch Environ Contam Toxicol 55: 33-42; doi: 10.1007/s00244-007-9097-y

197. Wani P.A., Wahid S., Khan M.S.A., Rafid N., Wahid N., 2019, Investigation of the role of chromium reductase for Cr (VI) reduction by Pseudomonas species isolated from Cr (VI) contaminated effluent, Biotechnology Research and Innovation 3: 38-46; doi: 10.1016/j.biori.2019.04.001

198. Weeger W., Lièvremont D., Perret M., Lagarde F., Hubert J.-C., Leroy M., Lett M.-C., 1999, Oxidation of arsenite to arsenate by a bacterium isolated from an aquatic environment, BioMetals 12: 141-149

199. Wittig Franco M., Aparecida Mendes L., Carvalhinho Windmöller C., Ferreira Moura K.A., Gomes Oliveira L.A., Rodrigues Barbosa F.A., 2018, Mercury Methylation Capacity and Removal of Hg Species from Aqueous Medium by Cyanobacteria, Water Air Soil Pollut 229: 127; doi: 10.1007/s11270-018-3782-5

200. Wright M.H., Geszvain K., Oldham V.E., Luther G.W., Tebo B.M.. 2018, Oxidative Formation and Removal of Complexed Mn(III) by Pseudomonas Species, Front. Microbiol. 9: 560.; doi: 10.3389/fmicb.2018.00560

201. Wu J., Hang F., Wang Z., Song L., Guan X., Zhou H., 2022b, Manganese removal and product characteristics of a marine manganese‑oxidizing bacterium Bacillus sp. FF‑1, International Microbiology 25: 701-708; doi: 10.1007/s10123-022-00254-9

202. Wu Y.-F., Chai C.-W., Li Y.-N., Chen J., Yuan Y., Hu G., Rosen B.P., Zhang J., 2021, Anaerobic As(III) Oxidation Coupled with Nitrate Reduction and Attenuation of Dissolved Arsenic by Noviherbaspirillum Species, ACS Earth Space Chem. 5: 2115-2123; doi: 10.1021/acsearthspacechem.1c00155

203. Xiu W., Guo H., Shen J., Liu S., Ding S., Hou W., Ma J., Dong H., 2016, Stimulation of Fe(II) Oxidation, Biogenic Lepidocrocite Formation, and Arsenic Immobilization by

Pseudogulbenkiania Sp. Strain 2002, Environ. Sci. Technol. 50(12): 6449-6458; doi: 10.1021/acs.est.6b00562

204. Yang F., Li J., Wang H., Xiao X., Bai R., Zhao F., 2023, Visible light induces bacteria to produce superoxide for manganese oxidation, Front. Environ. Sci. Eng. 17(2): 19; doi: 10.1007/s11783-023-1619-y

205. Yang J., Pan X., Zhao C., Mou S., Achal V., Al-Misned F.A., Golam Mortuza M., Gadd G.M., 2016, Bioimmobilization of Heavy Metals in Acidic Copper Mine Tailings Soil, Geomicrobiology Journal 33(3-4): 261-266; doi: 10.1080/01490451.2015.1068889

206. Yang Y., Deng R., Long P., Huang Z.,Ren B., Wang Z., 2023, Isolation and Identification of Arsenic-oxidizing Bacterium Pseudomonas sp. AO-1 and Its Oxidation Properties for As(III), Ecology and Environment 32(3): 619-626; doi: 10.16258/j.cnki.1674-5906.2023.03.019

207. Yao Y., Hu L., Li S., Zeng Q., Zhong H., He Z., 2020, Exploration on the bioreduction mechanisms of Cr(VI) and Hg(II) by a newly isolated bacterial strain Pseudomonas umsongensis CY-1, Ecotoxicology and Environmental Safety 201: 110850; doi: 10.1016/j.ecoenv.2020.110850

208. Yoon I.H., Chang J.S., Lee J.H., Kim K.-W., 2009, Arsenite oxidation by Alcaligenes sp. strain RS-19 isolated from arsenic-contaminated mines in the Republic of Korea, Environ Geochem Health 31: 109–117; doi: 10.1007/s10653-008-9170-0

209. Yu H., Leadbetter J.R., 2020, Bacterial chemolithoautotrophy via manganese oxidation, Nature 583: 453-458; doi: 10.1038/s41586-020-2468-5

210. Zahoor A., Rehman A., 2009, Isolation of Cr(VI) reducing bacteria from industrial effluents and their potential use in bioremediation of chromium containing wastewater, J Environ Sci 21(6): 814-820; doi: 10.1016/s1001-0742(08)62346-3

211. Zawadzka A.M., Crawford R.L., Paszczynski A.J., 2007, Pyridine-2,6-bis(thiocarboxylic acid) produced by Pseudomonas stutzeri KC reduces chromium(VI) and precipitates mercury, cadmium, lead and arsenic, BioMetals 20: 145–158; doi: 10.1007/s10534-006-9022-2

212. Zeng Q., Hu Y., Yang Y., Hu L., Zhong H., He Z., 2019, Cell envelop is the key site for Cr(VI) reduction by Oceanobacillus oncorhynchi W4, a newly isolated Cr(VI) reducing bacterium, J Hazard Mater 15(368): 149-155; doi: 10.1016/j.jhazmat.2019.01.031

213. Zeng Y., Chen Z., Lyu Q., Cheng Y., Huan C., Jiang X., Yan Z., Tan Z., 2023, Microbiologically induced calcite precipitation for in situ stabilization of heavy metals contributes to land application of sewage sludge, Journal of Hazardous Materials 441: 129866; doi: 10.1016/j.jhazmat.2022.129866

214. Zeroual Y., Moutaouakkil A., Blaghen M., 2001, Volatilization of Mercury by Immobilized Bacteria (Klebsiella pneumoniae) in Different Support by Using Fluidized Bed Bioreactor, Current Microbiology 43: 322-327; doi: 10.1007/s002840010310

215. Zhang J., Zhou W., Liu B., He J., Shen Q., Zhao F.-J., 2015, Anaerobic Arsenite Oxidation by an Autotrophic Arsenite-Oxidizing Bacterium from an Arsenic-Contaminated Paddy Soil, Environ Sci Technol 49(10):5956-64, doi: 10.1021/es506097c

216. Zhang J.H., Lion L.W., Nelson Y.M., Shuler M.L., Ghiorse W.C., 2002, Kinetics of Mn (II) oxidation by Leptothrix discophora SS1. Geochim. Cosmochim. Acta 66: 773-781; doi: 10.1016/S0016-7037(01)00808-0

217. Zhang W., Chen L., Liu D., 2012, Characterization of a marine-isolated mercury-resistant Pseudomonas putida strain SP1 and its potential application in marine mercury reduction, Appl Microbiol Biotechnol 93: 1305-1314; doi: 10.1007/s00253-011-3454-5

218. Zhang Y., Tang Y., Qin Z., Luo P., Ma Z., Tan M., Kang H., Huang Z., 2019, A novel manganese oxidizing bacterium-Aeromonas hydrophila strain DS02: Mn(II) oxidization and biogenic Mn

oxides generation, Journal of Hazardous Materials 367: 539-545; doi: 10.1016/j.jhazmat.2019.01.012

219. Zhang Z., Yin N., Cai X., Wang Z., Cui Y., 2016, Arsenic redox transformation by Pseudomonas sp. HN-2 isolated from arsenic-contaminated soil in Hunan, China, Journal of Environmental Sciences 47: 165-173; doi: 10.1016/j.jes.2015.11.036

220. Zhao M., Zheng G., Kang X., Zhang X, Guo J., Zhang M., Zhang J., Chen Y., Xue L., 2023, Arsenic pollution remediation mechanism and preliminary application of arsenic-oxidizing bacteria isolated from industrial wastewater, Environmental Pollution 324: 121384; doi: 10.1016/j.envpol.2023.121384

221. Zhao R., Wang B., Cai Q.T., Li X.X., Liu M., Hu D., Guo D.B., Wang J., Fan C., 2016, Bioremediation of hexavalent chromium pollution by Sporosarcina saromensis M52 isolated from offshore sediments in Xiamen, China. J. Biomed. Environ. Sci. 29: 127-136; doi: 10.3967/bes2016.014

222. Zhao X., Do H.T., Zhou Y., Li Z., Zhang X., Zhao S., Li M., Wu D., 2019, Rahnella sp. LRP3 induces phosphate precipitation of Cu (II) and its role in copper-contaminated soil remediation, Journal of Hazardous Materials 368: 133-140; doi: 10.1016/j.jhazmat.2019.01.029

223. Zhao X., Wang X., Liu B., Xie G., Xing D., 2018, Characterization of manganese oxidation by Brevibacillus at different ecological conditions, Chemosphere 205: 553–55, doi: 10.1016/j.chemosphere.2018.04.130

224. Zheng Z., Li Y., Zhang X., Liu P., Ren J., Wu G., Zhang Y., Chen Y., Li X., 2015, A Bacillus subtilis strain can reduce hexavalent chromium to trivalent and an nfrA gene is involved, International Biodeterioration & Biodegradation 97: 90e96, doi: 10.1016/j.ibiod.2014.10.017

225. Zhu X., Li W., Zhan L., Huang M., Zhang Q., Achal V., 2016, The large-scale process of microbial carbonate precipitation for nickel remediation from an industrial soil, Environmental Pollution 219: 149-155; doi: 10.1016/j.envpol.2016.10.047

226. Zou G., Xia X., Wang H., Li L., Wang G., Zheng S., Liao S., 2016, Immobilization of Lead by Alishewanella sp. WH16-1 in Pot Experiments of Pb-Contaminated Paddy Soil, Water Air Soil Pollut 227: 339; doi: 10.1007/s11270-016-3040-7
